# Effect of Delayed Targeted Intraoperative Radiotherapy vs Whole-Breast Radiotherapy on Local Recurrence and Survival: Long-term Results From the TARGIT-A Randomized Clinical Trial in Early Breast Cancer

**DOI:** 10.1001/jamaoncol.2020.0249

**Published:** 2020-04-02

**Authors:** Jayant S. Vaidya, Max Bulsara, Christobel Saunders, Henrik Flyger, Jeffrey S. Tobias, Tammy Corica, Samuele Massarut, Frederik Wenz, Steffi Pigorsch, Michael Alvarado, Michael Douek, Wolfgang Eiermann, Chris Brew-Graves, Norman Williams, Ingrid Potyka, Nicholas Roberts, Marcelle Bernstein, Douglas Brown, Elena Sperk, Siobhan Laws, Marc Sütterlin, Steinar Lundgren, Dennis Holmes, Lorenzo Vinante, Fernando Bozza, Montserrat Pazos, Magali Le Blanc-Onfroy, Günther Gruber, Wojciech Polkowski, Konstantin J. Dedes, Marcus Niewald, Jens Blohmer, David McCready, Richard Hoefer, Pond Kelemen, Gloria Petralia, Mary Falzon, Michael Baum, David Joseph

**Affiliations:** 1 Division of Surgery and Interventional Science, University College London, London, United Kingdom; 2 Department of Biostatistics, University of Notre Dame, Fremantle, West Australia, Australia; 3 University of Western Australia School of Surgery, West Australia, Australia; 4 Department of Breast Surgery, University of Copenhagen, Copenhagen, Denmark; 5 Department of Clinical Oncology, University College London Hospitals, London, United Kingdom; 6 Department of Radiation Oncology, Sir Charles Gairdner Hospital, Perth, West Australia, Australia; 7 Department of Surgery, Centro di Riferimento Oncologico di Aviano (CRO) IRCCS, Aviano, Italy; 8 University Medical Center Mannheim, Department of Radiation Oncology, Medical Faculty Mannheim, Heidelberg University, Germany; 9 Red Cross Hospital, Department of Gynecology and Obstetrics, Technical University of Munich, Munich, Germany; 10 Department of Surgery, University of California, San Francisco; 11 Nuffield Department of Surgical Sciences, University of Oxford, Oxford, United Kingdom; 12 Patient Advocate and Writer, London, United Kingdom; 13 Department of Surgery, Ninewells Hospital, Dundee, United Kingdom; 14 Department of Surgery, Royal Hampshire County Hospital, Winchester, United Kingdom; 15 University Medical Center Mannheim, Department of Gynecology and Obstetrics, Medical Faculty Mannheim, Heidelberg University, Germany; 16 Department of Oncology, St Olav’s University Hospital, Trondheim, Norway; 17 Department of Clinical and Molecular Medicine, Norwegian University of Science and Technology (NTNU), Trondheim, Norway; 18 Helen Rey Breast Cancer Foundation, John Wayne Cancer Institute, University of Southern California, Los Angeles; 19 Department of Radiation Oncology, Centro di Riferimento Oncologico di Aviano (CRO) IRCCS, Aviano, Italy; 20 Instituto Oncologico Veneto, Padoa, Italy; 21 University Hospital, Department of Radiation Oncology, Ludwig Maximilians Universitat, Munich, Germany; 22 Oncologue radiothérapeute, Institut de Cancérologie de l’Ouest, Nantes, France; 23 Brust Zentrum Seefeld, Zurich, Zurich, Switzerland; 24 Department of Surgical Oncology, Medical University of Lublin, Lublin, Poland; 25 Breast Center, Universitätsspital Zürich, Zurich, Switzerland; 26 Saarland University Medical Center, Homberg, Germany; 27 Sankt Gertrauden-Krankenhaus, and The Charité – Universitätsmedizin Berlin, Berlin, Germany; 28 Princess Margaret Cancer Centre Toronto, Toronto, Ontario, Canada; 29 Sentara Surgery Specialists, Hampton, Virginia; 30 Ashikari Breast Center, New York Medical College, New York, New York; 31 Department of Surgery, University College London Hospitals, London, United Kingdom; 32 Department of Pathology, University College London Hospitals, London, United Kingdom

## Abstract

**Importance:**

Conventional adjuvant radiotherapy for breast cancer given daily for several weeks is onerous and expensive. Some patients may be obliged to choose a mastectomy instead, and some may forgo radiotherapy altogether. We proposed a clinical trial to test whether radiotherapy could be safely limited to the tumor bed.

**Objective:**

To determine whether delayed second-procedure targeted intraoperative radiotherapy (TARGIT-IORT) is noninferior to whole-breast external beam radiotherapy (EBRT) in terms of local control.

**Design, Setting, and Participants:**

In this prospective, randomized (1:1 ratio) noninferiority trial, 1153 patients aged 45 years or older with invasive ductal breast carcinoma smaller than 3.5 cm treated with breast conservation were enrolled from 28 centers in 9 countries. Data were locked in on July 3, 2019.

**Interventions:**

The TARGIT-A trial was started in March 2000; patients were randomized after needle biopsy to receive TARGIT-IORT immediately after lumpectomy under the same anesthetic vs EBRT and results have been shown to be noninferior. A parallel study, described in this article, was initiated in 2004; patients who had their cancer excised were randomly allocated using separate randomization tables to receive EBRT or delayed TARGIT-IORT given as a second procedure by reopening the lumpectomy wound.

**Main Outcomes and Measures:**

A noninferiority margin for local recurrence rate of 2.5% at 5 years, and long-term survival outcomes.

**Results:**

Overall, 581 women (mean [SD] age, 63 [7] years) were randomized to delayed TARGIT-IORT and 572 patients (mean [SD] age, 63 [8] years) were randomized to EBRT. Sixty patients (5%) had tumors larger than 2 cm, or had positive nodes and only 32 (2.7%) were younger than 50 years. Delayed TARGIT-IORT was not noninferior to EBRT. The local recurrence rates at 5-year complete follow-up were: delayed TARGIT-IORT vs EBRT (23/581 [3.96%] vs 6/572 [1.05%], respectively; difference, 2.91%; upper 90% CI, 4.4%). With long-term follow-up (median [IQR], 9.0 [7.5-10.5] years), there was no statistically significant difference in local recurrence-free survival (HR, 0.75; 95% CI, 0.57-1.003; *P* = .052), mastectomy-free survival (HR, 0.88; 95% CI, 0.65-1.18; *P* = .38), distant disease-free survival (HR, 1.00; 95% CI, 0.72-1.39; *P* = .98), or overall survival (HR, 0.96; 95% CI, 0.68-1.35; *P* = .80).

**Conclusions and Relevance:**

These long-term data show that despite an increase in the number of local recurrences with delayed TARGIT-IORT, there was no statistically significant decrease in mastectomy-free survival, distant disease-free survival, or overall survival.

**Trial Registration:**

ISRCTN34086741, ClinicalTrials.gov Identifier: NCT00983684

## Key Points

**Question:**

For early breast cancer, is 5-year local control with delayed second-procedure targeted intraoperative radiotherapy (TARGIT-IORT) noninferior to whole-breast postoperative external beam radiotherapy (EBRT), and how do long-term outcomes compare?

**Findings:**

In this randomized clinical trial including 1153 participants, delayed second-procedure TARGIT-IORT was not noninferior to EBRT at 5-year complete follow-up; however, long-term (median 9 years) mastectomy-free survival, distant disease-free survival, and overall survival were not different.

**Meaning:**

For early breast cancer, delayed second-procedure single-dose TARGIT-IORT given by reopening the lumpectomy wound had similar long-term mastectomy-free and overall survival compared with EBRT despite higher local recurrence.

## Teaser

This secondary analysis of the TARGIT-A randomized clinical trial compares the effect of delayed second-procedure targeted intraoperative radiotherapy (TARGIT-IORT) with whole-breast external beam radiotherapy (ERBT) on local recurrence at 5 years and long-term survival outcomes among women with early breast cancer.

## Introduction

In 2018, there were 2 million new cases of breast cancer diagnosed worldwide and 626 000 deaths.^1^ Most patients are suitable for treatment with breast-conserving surgery and adjuvant radiotherapy, rather than total mastectomy. The TARGIT-A randomized clinical trial (accrual from 2000-2012) compared risk-adapted TARGeted intraoperative radiotherapy (TARGIT-IORT) during the initial surgical excision of the cancer^2,3,4,5^ with conventional whole-breast external beam radiotherapy (EBRT) over several weeks.^2,6,7^ The results of this trial demonstrated noninferiority particularly when TARGIT-IORT was delivered at the time of initial excision of cancer.

In 2004, 4 years after accrual began in the main TARGIT-A trial, and at the request of potentially high-volume centers, we sought and received additional ethics approval and opened a parallel study. This was previously referred to as “postpathology stratum” and recruited 1153 patients using a separate randomization table. Patients were randomized after their initial surgery to have either conventional fractionated whole-breast radiotherapy (n = 572), or to undergo a further operation to deliver delayed radiotherapy to the wound (n = 581) by reopening the original incision. This trial was initiated mainly because of the convenience of easier scheduling of delayed TARGIT-IORT in the operation theater. A potential benefit was that the inclusion criteria could be made more selective, choosing the patients with better prognosis based on the full histopathologic results that would be available after tumor excision. For example, the knowledge of the microscopically measured tumor size, grade, and nodal status could be used to select a much lower-risk patient population before randomization.

This delayed procedure was performed at a median (IQR) of 37 (29-51) days after the initial excision as a second surgical procedure in the operation theater, rather than immediate intraoperative radiotherapy given during the initial cancer operation. This article describes the long-term outcomes of this parallel study.

## Methods

The TARGIT-A trial was a pragmatic, prospective, international, multicenter, open label, randomized, phase 3 trial that compared the policy of risk-adapted TARGIT-IORT vs the conventional policy of whole-breast EBRT. The trial protocol (https://njl-admin.nihr.ac.uk/document/download/2006598) and the details of sample size calculations, the process of random allocation, have been previously described.^6,7^ The trial protocol is available in Supplement 1. The study received ethics approval from the joint University College London and University College London Hospital committees of ethics of human research.

### Participants

**Figure 1.**
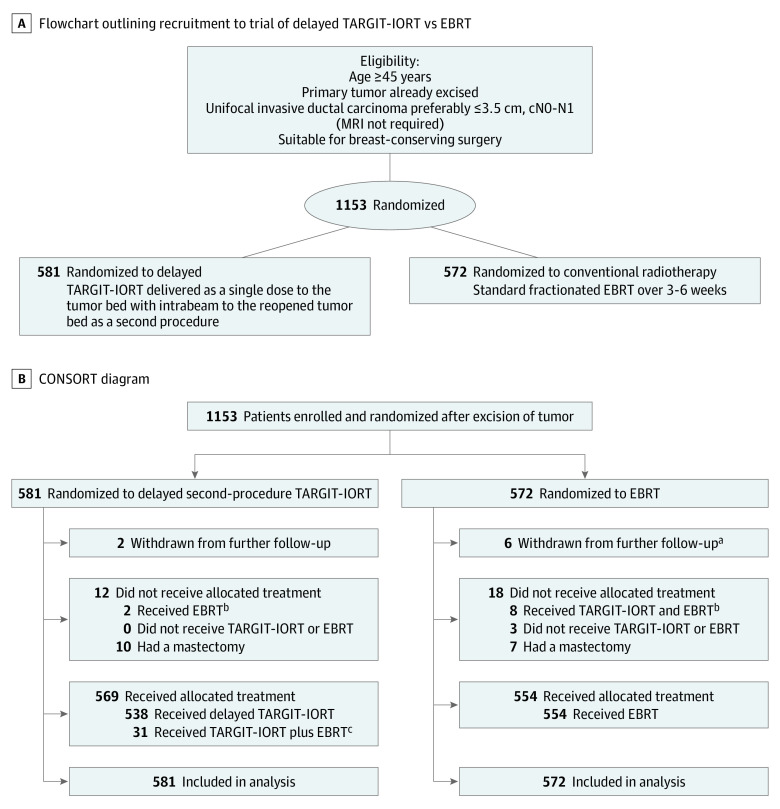
Flowchart and CONSORT Diagram EBRT indicates whole-breast external beam radiotherapy; MRI, magnetic resonance imaging; TARGIT-IORT, targeted intraoperative radiotherapy. A, Flowchart outlining recruitment to trial of delayed TARGIT-IORT vs EBRT. B, CONSORT diagram of participant randomization. ^a^The difference in number withdrawn was not statistically significant (*P* = .15). ^b^As per protocol, 31 of 581 patients (5.3 %) allocated to delayed TARGIT-IORT received EBRT after TARGIT-IORT. ^c^Two of 581 patients (0.3%) allocated to delayed TARGIT-IORT received EBRT and 8 of 572 (1.4%) allocated EBRT received TARGIT-IORT as well.

Women were eligible to participate in the delayed TARGIT-IORT trial if their breast cancer was already excised. They needed to be aged 45 years or older with unifocal breast cancer on examination and conventional imaging. Pragmatically, we permitted individual centers to prespecify the final postoperative histopathologic criteria that would make patients eligible for randomization and these were prespecified in the center’s treatment policy document. Because most centers specified criteria for eligibility: aged 50 years or older, grade 1 or 2 disease, and uninvolved nodes, only 5% of patients in the trial had any adverse prognostic criteria. All patients gave informed written consent and needed to be available for regular follow-up for at least 10 years. Follow-up clinical examination was at least every 6 months for the first 5 years and annually thereafter, including a mammogram once per year. Random allocation was in a 1:1 ratio, to receive either single-dose delayed TARGIT-IORT or EBRT as per standard schedules over several weeks, with randomization blocks stratified by center. The flow diagram and CONSORT diagram are given in Figure 1A and B.

The concept and the delayed TARGIT-IORT technique have been described previously^3,4,5,8,9,10,11^ and enabled these patients to have their radiotherapy in 1 sitting, albeit by undergoing a second procedure, usually under a general anesthetic.^12^ Radiation was given over 20 to 50 minutes delivering 20 Gy to the surface of the tumor bed attenuating to 5 to 7 Gy at 1-cm depth.

The patients in the conventional arm underwent standard EBRT, which always included fractionated whole-breast radiotherapy for 3 to 6 weeks, with or without an EBRT tumor bed boost, as determined by local criteria prespecified by the collaborating center.

### Statistical Analysis

The statistical analysis plan (Supplement 1) was signed off on by the chair of the independent steering committee and an independent senior statistician before the unblinded data were sent to the trial statistician for the current analysis. It specified the primary outcome as local recurrence-free survival. This outcome, consistent with the DATECAN^13^ and STEEP^14^ guidelines, estimates the chance of a patient being alive without local recurrence and therefore included local recurrence or death as events, ie, patients who had died were not censored. The other outcomes included mastectomy-free survival, distant disease-free survival, overall survival, breast cancer mortality and non–breast cancer mortality. Statistical analysis was performed using established methods, using STATA statistical software (versions 15.0 and 16.0, STATA Corp) for data compilation, validation, and analysis.^13,14,15^ Data analysis took place between September 11, 2019 to January 15, 2020.

In the original protocol, noninferiority was specified as being achieved if the difference in 5-year local recurrence rate did not cross a stringent margin of 2.5%. However, we have applied an even more rigorous criterion since 2013: that the upper 90% CI of the absolute difference in the binomial proportions of local recurrence rate at 5-year complete follow up should not cross 2.5% in absolute terms.

Kaplan-Meier graphs were displayed as recommended by Pocock et al,^16^ who recommend that the x-axis of these graphs should be extended until 10% to 20% of patients are at risk of an event. The log-rank test was used to compare the difference between survival functions and to obtain *P* values.

### Main Outcomes and Measures

The cause of death was specified by the center. If the cause was specified as a non–breast cancer event and no distant disease was recorded, it was defined as a non–breast cancer death. If the death was recorded by the center to be related to breast cancer, or as per convention, if breast cancer was present at the time of death, or if the cause of death was recorded as unknown or uncertain, it was presumed to be a breast cancer death.

Figure 1B shows the CONSORT diagram, which describes the treatment received in each of the randomized arms. The reference date for completeness was May 2, 2018, 8 years after the first data lock. A patient was considered as having complete follow-up if they were seen for the specified duration of follow-up, had died, or had withdrawn from the trial. As the last patient was randomized in 2012, the statistical analysis plan specified that the 5-year follow-up would be considered complete if 95% of patients had complete follow-up. It also specified that 10-year follow-up would be considered complete if the patient had at least 10 years of follow-up, had been seen within 1 year of the reference date, or had died or withdrawn; the 10-year follow-up would be considered complete if this was achieved by 90% of patients. Because there was no specific trial funding for individual centers, return of follow-up relied on individual investigators and their teams’ efforts, enthused by the trial-center team. The trial statistician and the chief investigator produced reports of completeness of follow up using blinded databases on a regular basis. As recommended by the independent steering committee, the database was unblinded for analysis once the prespecified goals for completeness of follow up were achieved. The reference date for analysis was 3 July 2019, so that all events up until 2 July 2019 were included for analysis. The chief investigator/corresponding author and the trial statistician (J.S.V. and Ma.B.) had access to all data sent by the trial center for analysis; all authors were responsible for the decision to submit the article. Since the last analysis, the trial oversight has been provided by an independent steering committee, appointed by the Health Technology Assessment program of the National Institute of Health Research, Department of Health, United Kingdom.

## Results

**Figure 2.**
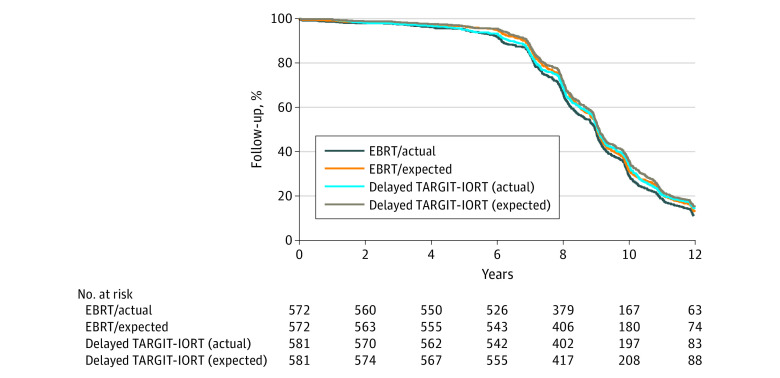
Actual Follow-up and Expected Follow-up for the Trial of Delayed Second-Procedure TARGIT-IORT vs EBRT EBRT indicates whole-breast external beam radiotherapy; TARGIT-IORT, targeted intraoperative radiotherapy.

Overall, 581 women were randomized to delayed TARGIT-IORT and 572 to EBRT. The patient and tumor characteristics are given in Table 1 and were well matched between the randomization arms. Most patients were estrogen receptor positive (1119 [98%]), *ERBB2* negative (1041 [94%]); 670 patients (58%) received endocrine therapy, and 40 (3.5%) received chemotherapy. The completeness of follow-up is demonstrated in Figure 2.

**Table 1.** Patient and Tumor Characteristics

Characteristic	No. (%)^a^		*P* value^b^
	Delayed TARGIT-IORT (n = 581)	EBRT (n = 572)	
Age, y			
≤50	30 (5.2)	23 (4.02)	.54
51-60	166 (28.6)	171 (29.9)	
61-70	302 (52.0)	284 (49.7)	
>70	83 (14.3)	94 (16.4)	
Pathologic tumor size, mm			
≤10	294 (51.0)	290 (51.8)	.79
11-20	249 (43.2)	243 (43.4)	
>20	33 (5.7)	27 (4.8)	
Unknown	5 (0.9)	12 (2.1)	
Grade			
1	305 (56.5)	339 (63.8)	.06
2	204 (37.8)	159 (29.9)	
3	31 (5.7)	33 (6.2)	
Unknown	41 (7.1)	41 (7.2)	
Margin			
Free	539 (92.9)	520 (92.4)	.46
DCIS only	16 (2.8)	18 (3.2)	
Invasive	25 (4.3)	25 (4.5)	
Unknown	1 (0.2)	9 (1.6)	
Lymphovascular invasion			
Absent	536 (94.7)	533 (96.6)	.13
Present	30 (5.3)	19 (3.4)	
Unknown	15 (2.6)	20 (3.5)	
Lymph nodes involved			
0	543 (93.6)	537 (95.2)	.39
1-3	34 (5.9)	26 (4.6)	
>3	3 (0.5)	1 (0.2)	
Unknown	1 (0.2)	8 (1.4)	
ER status			
Positive	569 (98.3)	550 (97.9)	.62
Negative	10 (1.7)	12 (2.1)	
Unknown	2 (0.3)	10 (1.7)	
PgR status			
Positive	440 (81.8)	423 (82.0)	.94
Negative	98 (18.2)	93 (18.0)	
Unknown	43 (7.4)	56 (9.8)	
*ERBB2* status			
Positive	30 (5.4)	33 (6.0)	.65
Negative	526 (94.6)	515 (94.0)	
Unknown	25 (4.3)	24 (4.2)	
Method of presentation			
Screen detected	420 (73.6)	395 (70.5)	.26
Symptomatic	151 (26.4)	165 (29.5)	
Unknown	10 (1.7)	12 (2.1)	
Endocrine therapy			
Received	336 (58.0)	334 (59.4)	.63
Did not receive	243 (42.0)	228 (40.6)	
Unknown	2 (0.3)	10 (1.8)	
Chemotherapy			
Received	26 (4.5)	14 (2.5)	.07
Did not receive	553 (95.5)	546 (97.5)	
Unknown	2 (0.3)	12 (2.1)	

Abbreviations: DCIS, ductal carcinoma in situ; EBRT, whole-breast external beam radiotherapy; ER, estrogen receptor; PgR, progesterone receptor; TARGIT-IORT, targeted intraoperative radiotherapy.

^a^ For percentage calculation, the denominator for unknown percentages is the total number randomized (581 and 572) and the denominator for each category is the total number of known cases.

^b^ *P* values are given for differences between TARGIT-IORT and EBRT, calculated using a χ^2^ test for known values.

At 5-year complete follow-up, the local recurrence rates were TARGIT-IORT, 23 (including 3 DCIS) of 581 (3.96%) vs EBRT, 6 (including 2 DCIS) of 572 (1.05%), giving a difference of 2.9% with its upper 90% CI of 4.4, which crossed the noninferiority margin of 2.5%.

**Figure 3.**
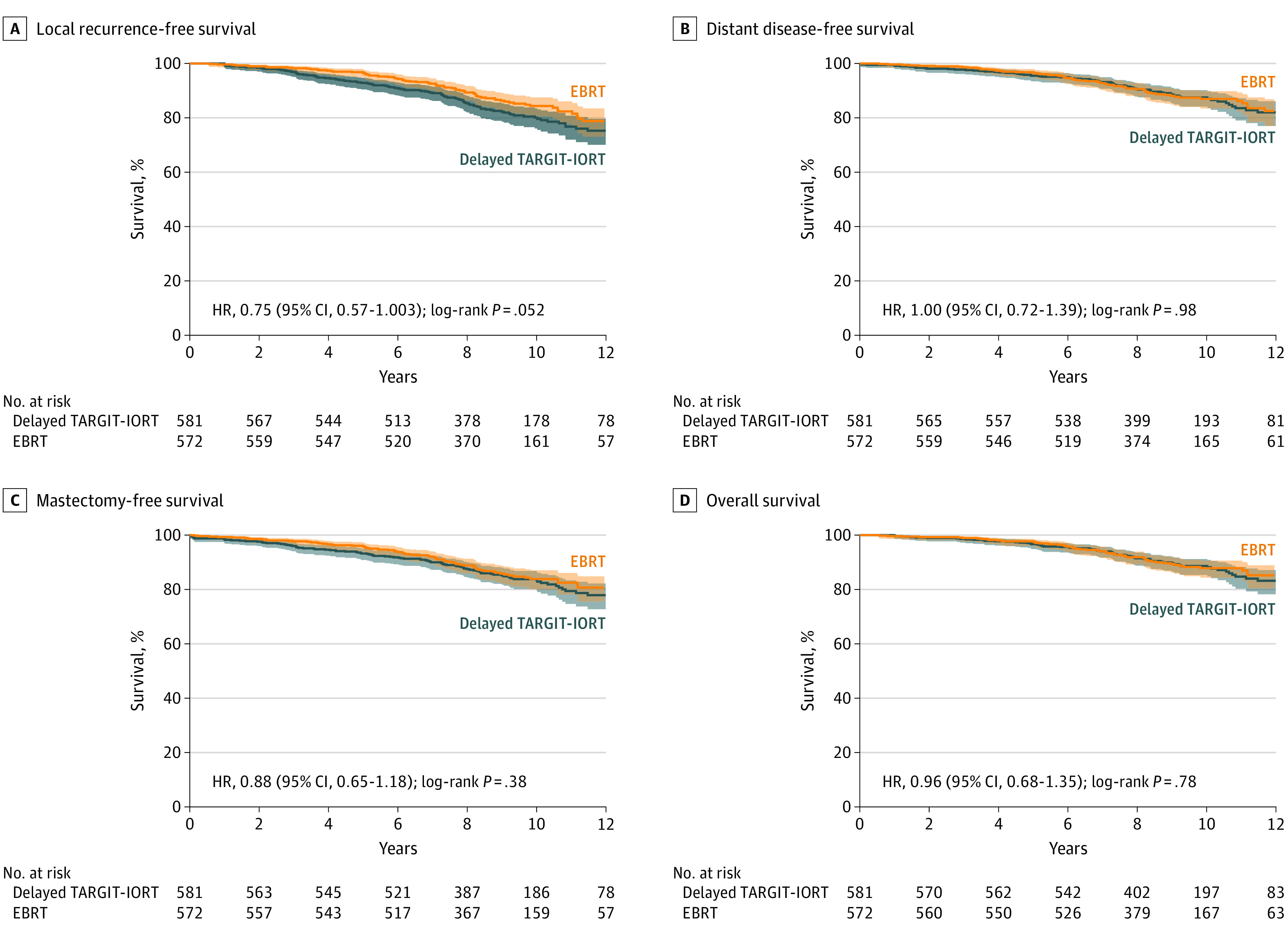
Twelve-Year Kaplan-Meier Curves Comparing Delayed Second-Procedure TARGIT-IORT vs EBRT EBRT indicates whole-breast external beam radiotherapy; TARGIT-IORT, targeted intraoperative radiotherapy. In each of these Kaplan-Meier graphs, the blue lines represent delayed TARGIT-IORT with light blue shading indicating the 95% confidence intervals. The orange lines represent EBRT with light orange shading indicating the 95% confidence intervals.

Kaplan-Meier estimates and log-rank *P* values for delayed TARGIT-IORT vs EBRT are given in Table 2 and Figure 3. The median follow-up was 9 years and the differences between delayed TARGIT-IORT and EBRT were not statistically significant for local recurrence-free survival, invasive local recurrence-free survival, mastectomy-free survival, distant disease-free survival, breast cancer mortality, non–breast cancer mortality, and overall survival. No patients had uncontrolled local recurrence at the time of death.

**Table 2.** Twelve-Year Kaplan-Meier Estimates of Outcomes Measures for TARGIT-IORT vs EBRT

Outcomes	Delayed TARGIT-IORT (n = 581)		EBRT (n = 572)		Significance test for the full follow-up	
	Events	Kaplan-Meier estimates (95% CI)	Events	Kaplan-Meier estimates (95% CI)	HR (95% CI)	*P* value for log rank
**Local recurrence-free survival**^a^						
Estimate					0.75 (0.57-1.003)	.052
5-y	41	92.87 (90.44-94.70)	19	96.63 (94.77-97.84)		
10-y	98	80.16 (76.19-83.54)	72	84.36 (80.51-87.51)		
12-y	106	75.30 (70.13-79.72)	79	78.38 (72.32-83.27)		
**Invasive local recurrence-free survival**^a^						
Estimate					0.75 (0.56-1.002)	.051
5-y	38	93.39 (91.03-95.15)	17	96.99 (95.20-98.12)		
10-y	95	80.68 (76.73-84.02)	68	85.15 (81.35-88.23)		
12-y	103	75.87 (70.72-80.24)	75	79.23 (73.23-84.04)		
**Mastectomy-free survival**^a^						
Estimate					0.88 (0.65-1.18)	.38
5-y	39	93.24 (90.87-95.02)	23	95.93 (93.93-97.27)		
10-y	82	83.79 (80.14-86.83)	75	83.82 (79.94-87.01)		
12-y	92	77.80 (72.57-82.16)	79	80.44 (75.16-84.71)		
**Distant disease-free survival**^a^						
Estimate					1.00 (0.72-1.39)	.98
5-y	26	95.49 (93.44-96.90)	18	96.80 (94.97-97.97)		
10-y	62	87.50 (84.13-90.19)	62	86.91 (83.37 89.74)		
12-y	71	81.98 (76.91-86.04)	67	82.18 (76.44-86.65)		
**Overall survival **						
Estimate					0.96 (0.68-1.35)	.80
5-y	19	96.70 (94.87-97.88)	13	97.69 (96.06-98.65)		
10-y	56	88.62 (85.35-91.19)	56	87.77 (84.22-90.56)		
12-y	65	83.13 (78.11-87.10)	59	84.72 (79.52-88.70)		
**Breast cancer mortality **						
Estimate					0.81 (0.43-1.52)	.50
5-y	9	1.58 (0.82-3.01)	4	0.72 (0.27-1.90)		
10-y	20	3.79 (2.45-5.83)	16	3.50 (2.11-5.77)		
12-y	21	4.39 (2.77-6.93)	17	4.63 (2.52-8.43)		
**Mortality from other causes **						
Estimate					1.02 (0.68-1.55)	.89
5-y	10	1.75 (0.95-3.23)	9	1.60 (0.84-3.06)		
10-y	36	7.90 (5.69-10.90)	40	9.05 (6.62-12.31)		
12-y	44	13.05 (9.35-18.05)	42	11.17 (7.78-15.88)		

Abbreviations: EBRT, whole-breast external beam radiotherapy; HR, hazard ratio; TARGIT-IORT, targeted intraoperative radiotherapy.

^a^ Each of these survival measures include death as an event.

## Discussion

The TARGIT-A trial was originally conceived because of the clinicopathologic observation that local recurrence after breast-conserving surgery occurs predominantly in the index quadrant,^17,18^ despite the fact that more than 60% of patients suitable for breast conserving surgery are known to have microscopic foci of the disease outside the index quadrant.^17,18,19^

The delayed TARGIT-IORT approach was proposed mainly for logistical reasons. It allowed better planning of operation theaters as well as theoretically stricter selection of patients with low-risk disease based on final histopathologic analysis results. It also allowed using TARGIT-IORT in patients coming to a cancer center after having had their cancer excised in a smaller or remote hospital. Concordant with the results of our 2013 analysis, with mature follow-up (5 years complete follow-up with a median of 9 years) delayed TARGIT-IORT was found not to be noninferior to EBRT in terms of local control, with the upper 90% confidence limit of the 2.9% absolute difference in the 5-year local recurrence rate being 4.4%, which is above our stringent 2.5% noninferiority margin.

This noninferiority margin of 2.5% was decided after considerable thought,^6^ and is much more stringent than the 7% margin set in the in the ELIOT trial, the only other trial to our knowledge of intraoperative radiotherapy.^20^ We believe that it is important to consider how much the absolute differences seen in the trial matter to the patient. When considering treatments for patients with early breast cancer, local recurrence has been given great importance because of the perceived risk of consequent mastectomy, the danger of distant disease, and the potentially lower survival. The long-term data show that there was no impairment of mastectomy-free survival, distant disease-free survival, or overall survival, up to 12 years from randomization (Figure 3). Moreover, quality of life studies have shown that despite having a second procedure, the quality of life and patient-reported outcomes, such as cosmesis, breast-related quality of life, and breast pain, have been demonstrated to be superior with TARGIT-IORT,^21,22^ and this approach is preferred by patients even in the face of a hypothetically higher local recurrence risk.^23,24^ These findings may mitigate some of the patient concerns, and results of further patient preference research would help these discussions.

### Limitations

The reasons for higher local recurrence with delayed second-procedure TARGIT-IORT may be multifactorial. First, the propensity of tumor recurrence in the index quadrant could be owing to a tumor promoting effect of the microenvironment of the surgical wound,^25,26,27^ a risk that has been shown to be beneficially manipulated by TARGIT-IORT to the fresh tumor bed,^25,27,28^ but perhaps not when TARGIT-IORT is given as a delayed second procedure. Second, the surgical procedure of lumpectomy has changed. Early on in the trial, the tissues around the tumor bed were often not approximated after lumpectomy, and the tumor bed remained easily identifiable as a fluid-filled cavity at the time of the second procedure, although some healing had already occurred and fibrosis was setting in by the time the delayed TARGIT-IORT was delivered (median, 37 days later). A limitation of the study was that we did not anticipate a change in surgical practice in later years, such that the tumor bed was approximated after tumor excision rather than leaving a cavity. The resultant scarring could have made it difficult to accurately locate the primary tumor bed. Given the rapid attenuation of dose, with distance from the applicator surface, adequate dose may not have reached the original tumor bed. Finally, one can also speculate that the additional surgical trauma owing to the necessary second procedure in every case of delayed TARGIT-IORT could stimulate residual cancer cells. Notwithstanding these theoretical reasons, the final judgments must be based on the long-term outcomes data.

## Conclusions

Partial breast irradiation was heralded as a new standard^29^ at the time of the first publication of the TARGIT-A trial^6^ and several other supporting clinical trials have since been published: including the ELIOT trial,^20^ interstitial wire-brachytherapy,^30^ and partial breast EBRT.^31,32^ Based on the randomized evidence of immediate TARGIT-IORT, which has been shown to be an effective alternative to EBRT,^6,7,33^ it is clear that the preferred timing of using TARGIT-IORT is immediately—during the initial surgical excision of breast cancer. However, when immediate TARGIT-IORT has not been possible, the long-term data presented in this article may help inform discussions by clinicians and patients who wish to avoid a prolonged postoperative course of EBRT.

## Notes

Supplement 1. Trial Protocol

Supplement 2. Data Sharing Statement
