# Supplementary material for: Effect of Delayed Targeted Intraoperative Radiotherapy vs Whole-Breast Radiotherapy on Local Recurrence and Survival: Long-term Results From the TARGIT-A Randomized Clinical Trial in Early Breast Cancer
Source: JAMA Oncol. 2020 Apr 2;6(7):e200249. doi: 10.1001/jamaoncol.2020.0249 (PMC7348682; doi:10.1001/jamaoncol.2020.0249)
Supplement: Supplement 1. — Trial Protocol [file jamaoncol-6-e200249-s001.pdf]

SAP (2019)

Statistical Analysis Plan for the TARGIT-A Trial

_TARG_eted _I_ntraoperative radio_T_herapy

vs.

Post Operative Radiotherapy

An international randomised controlled trial to compare targeted intra-operative radiotherapy with conventional post-operative radiotherapy after conservative breast surgery for women with early stage breast cancer

Statistical Analysis Plan Version 3.0
12 June 2019

TARGIT-A trial

ISRCTN 34086741
MREC No. 99/0307
UKCRN ID 7265
HTA - 07/60/49
ClinicalTrials.gov Identifier: NCT00983684

2 Signature Page

Statistical Analysis Plan for protocol:

TARGIT Trial: An international randomised controlled trial to compare targeted intra-operative radiotherapy with conventional post-operative radiotherapy after conservative breast surgery for women with early stage breast cancer. Version 4.0

Sponsor: University College London

Date: 12 June 2019

Status: DRAFT v2.0

Authors: Jayant S Vaidya, Max Bulsara, Norman Williams, Michael Baum

Approved by:

Trial Statistician

Professor Max Bulsara
Chair in Biostatistics
Institute of Health and Rehabilitation Research, University of Notre Dame,
19 Mouat Street, P.O Box 1225, Fremantle, WA 6959

Signature... Date: **14th June 2019**

Independent Steering Committee Chair

Professor Freddie Hamdy
Professor of Surgery
University of Oxford
Oxford

Signature... Date 14/6/2019

Professor Martin Bland
Professor of Health Statistics
Department of Health Sciences, Seebohm Rowntree Building
University of York, Heslington
York YO10 5DD
UK

Signature... Date ....

2 Signature Page

Statistical Analysis Plan for protocol:

TARGIT Trial: An international randomised controlled trial to compare targeted intra-operative radiotherapy with conventional post-operative radiotherapy after conservative breast surgery for women with early stage breast cancer. Version 4.0

Sponsor: University College London

Date: 12 June 2019

Status: DRAFT v2.0

Authors: Jayant S Vaidya, Max Bulsara, Norman Williams, Michael Baum

Approved by:

Trial Statistician

Professor Max Bulsara
Chair in Biostatistics
Institute of Health and Rehabilitation Research, University of Notre Dame,
19 Mouat Street, P.O Box 1225, Fremantle, WA 6959

Signature.. Date: **14th June 2019**

Independent Steering Committee Chair

Professor Freddie Hamdy
Professor of Surgery
University of Oxford
Oxford

Signature.. Date ....

Professor Martin Bland
Professor of Health Statistics
Department of Health Sciences, Seebohm Rowntree Building
University of York, Heslington
York YO10 5DD
UK

Signature.. Date .... 29 June 2019

3 Version history

The first unblinded analysis of the TARGIT Trial took place in 2010 using a reference date of 02/05/2010, on the recommendation of the DMC. The emphasis was on efficacy and early safety. The results have been published (Lancet 2010; 376: 91-102).

The second unblinded analysis took place in 2012 using a reference date of 01/06/2012, on the recommendation of the DMC in 2010 (two years after the first analysis). The emphasis was on efficacy, and mortality. The results have been published (Lancet 2014; 383: 603–13).

This SAP is for a third unblinded analysis due to take place in 2019 using a reference date of 02/05/2018 on the recommendation of the TSC (completeness of follow-up at least 95% at 5 years and 90% at 10 years). The emphasis will be on all primary and secondary endpoints and subgroup analyses.

4 Table of Contents

1 Title page.. 1
2 Signature Page.. 2
3 Version history 3
4 Table of Contents 4
5 Study title 5
6 Study population 5
7 Objectives.. 5
7.1 Main Objective 5
7.2 Secondary Objectives.. 5
8 Statistical analysis plan 6
8.1 Preamble 6
8.2 Methodology 7
8.3 Statistical Software 7
9 Demographic and Baseline Characteristics 8
10 Outcomes 8
11 REFERENCES .... **Error! Bookmark not defined.**

5 Study title

TARGIT Trial: An international randomised controlled trial to compare targeted intra-operative radiotherapy with conventional post-operative radiotherapy after conservative breast surgery for women with early stage breast cancer

6 Study population

The study population has been described previously. There are two main strata in the trial. The prepathology stratum includes all patients who were randomised before their cancer was excised. The postpathology stratum includes all patients in whom the tumour had already been excised before randomisation so that for those randomised to TARGIT, re-opening of the wound was required to give targeted intraoperative radiotherapy. As the treatment delivery is so different between these two strata, and as they had separate randomisation tables, these were in fact two trials in parallel and therefore, all results will be presented separately for each stratum.

7 Objectives

7.1 Main Objective

To compare within each stratum, a single fraction of radiotherapy given intra-operatively and targeted to the tissues at the highest risk of local recurrence within a risk-adapted approach with standard post-operative radiotherapy after breast conserving surgery in women with early stage breast cancer in terms of survival without local recurrence in the conserved breast.

7.2 Secondary Objectives

To compare the treatment arms in each stratum with respect to:

- Overall survival (Breast Cancer and Non-Breast-Cancer Survival)
- Disease free survival
- Invasive-disease free survival
- Mastectomy and re-excision rate
- Treatment of local recurrence
- Site of relapse within the breast
- Local toxicity/morbidity

All outcomes in the following subgroups

- Effect of ER and PgR status on the outcomes
- Screen-detected vs symptomatic
- Effect of use of hormone therapy.
- Effect of Grade, node positivity, HER2 receptor status, etc.
- Effect of Centre and/ or Country on the outcomes
- Effect of all other predictive factors on the outcomes
- Re-do the relevant previously performed analyses with updated follow up information
- Outcomes as per treatment received along with subgroup analyses to refine understanding of what happens in the real world.

Additional analysis including Health economic analysis and cosmetic outcome, patient preference may also be undertaken.

8 Statistical analysis plan

8.1 Preamble

The statistical analysis plan for the analysis performed in 2012-13 stated that the major endpoint would be non-inferiority of local control with a non-inferiority margin of 2.5% at 5 years, for the whole trial and the Prepathology and Postpathology strata.

We have since learnt that non-inferiority analyses are ill understood and controversial. Although our 2.5% margin has been ratified in patient preference studies, it has been recognised as rather arbitrary; for example, the only other randomised trial (ELIOT) trial {Veronesi, 2013 #3853} of intraoperative radiotherapy for breast cancer used a non-inferiority margin of 7%. Two recent papers published in the Lancet on 6-month vs 12-month duration of adjuvant trastuzumab therapy found almost exactly the same results, but diametrically opposite conclusions and contradictory recommendations for practice simply because they had different non-inferiority margins [correspondence in post].

Furthermore, in the last few years, there was been a wide consensus that using p values for assessment of difference between outcomes can be misleading because of misattribution of a clinical significance to statistical significance of the difference. With increasingly large numbers of patients in clinical trials, you can reach the point where “statistical significance” does not correlate with “clinical significance”.

A substantial change in doctor-patient relationship has occurred in the last few years around the world. Unlike the paternalistic approach in which “clinical significance” was judged by the clinician on behalf of the patient, it is now well accepted that this ultimately needs to be judged by the well-informed patient. This assertion has been determined to be both ethically and legally mandated by the UK Supreme Court’s Montgomery judgement[1]

Finally, effect of radiotherapy on controlling local recurrence compared with no radiotherapy in long term follow up randomised clinical trials plateaus at 5 years and no additional difference arises between 5 and 20 years[2 3], both clinician and patient can be confident that the absolute difference in local recurrence is highly unlikely to change after the first 5 years.

In the name of probity and transparency we will conduct the statistical analyses in the ways we’ve published in the past but promote the importance of absolute numbers (not relative risks), and emphasise the actual values for the outcomes in each of the randomised arms, that matter to the patient and facilitate their decision-making process. The patient needs to know her chance of being alive without local recurrence – so local recurrence free survival is the relevant statistic, rather than the estimate of local recurrence calculated after censoring patients at the point of their death - which would give the artificial estimate as if patients live forever.

8.2 Methodology

The two arms of the trial will be compared on the basis of 'intention to treat': The methods below have been used in the previous analysis[4-25]

1. Non-inferiority will be tested using the difference in binomial proportions of local recurrence in the first 5 years and its 90% confidence interval using standard methods.
2. We shall also test for any difference in survival function using the log-rank test and Wilcoxon tests, and Kaplan-Meier curves and proportional hazard regression models will be used to account for time-to event and censoring of the data; however, these shall not be used for testing non-inferiority.
3. We shall also use the integrated difference of the two survival functions to quantify the difference between the Kaplan-Meier curves for local recurrence and local recurrence free survival [26]
4. In order to reduce the assumption of normal distribution in the above models, we shall also use the recently described method of restricted mean survival time approach for analysis of all outcomes [27-30]

For the primary end point of survival without local recurrence, given the difficulties in understanding the concept of non-inferiority and the criticism that the 2.5% margin may be considered arbitrary, we will describe the main results in terms of the absolute number of events as well as 5-year Kaplan-Meier estimate (and its 95% CI) of local recurrence and local recurrence free survival for each of the randomised arms of the trial.

For the analysis of overall survival, breast cancer survival and non-cancer survival, we shall report the absolute number of events, 5-year KM estimate as well as the analyses to test the null hypothesis of equality.

Statistical significance will be defined as per the current alpha spend. If p values are used, then, for local recurrence statistical significance will be at $p<0.001$ (using the Lan-DeMets spending function method) (it was 0.01 at the last analysis), and for survival it will be $p<0.025$ (it was 0.05 at the last analysis).

We shall also consider performing these analyses using the Bayesian methods using meaningful priors from previous analyses.

We shall transparently display the results of all analysis.

The baseline comparisons between the two groups will be done using chi-squared test for categorical variables and independent two sample t-test for all continuous variables.

8.3 Statistical Software

All data summaries and listings were performed using appropriate software as specified by the statistician (e.g., STATA)

9 Demographic and Baseline Characteristics

Demographic and baseline characteristics will be tabulated (see Appendix 1). In addition, if available, individual risks for non-breast-cancer morbidity and mortality will be compared [e.g. age, height, weight].

10 Outcomes

Completeness

1.1 A patient is considered to have complete follow up if they **are dead or withdrawn.** "Withdrawn" means the patient has withdrawn consent for further follow-up information to be collected.

1.2 A patient is considered as having **recent follow up** if their status is known within 1 year before the reference date for completeness (2 May 2018),

1.3 Completeness is assessed at 3 time points

1.3.1 5-years – the usual time to report

1.3.2 10-years – protocol specifies at least 10 year follow up of every patient.

Thus, a patient is considered to have

5-year complete follow up if they have at least 5 years of follow up or have a recent follow up or are dead/withdrawn – this was 93.5% at the time of the last publication

8-year complete follow up if they have at least 8 years of follow up or have a recent follow up or are dead/withdrawn

10-year complete follow up if they have at least 10 years of follow up or have a recent follow up or are dead/withdrawn

Completeness is judged by the following criteria by the TSC

1. Proportion with complete follow up should be high in each stratum
2. Patients' death is a major cause of not attending the clinic – and hence absence of status of follow up. Therefore, the mortality rate of a centres/countries should be similar to that of the gold standard centres as it would mean that the follow up is complete -

RD = reference date for completion of follow up, nominally 02/05/2018
N_5 = number of patients with at least five years of follow-up (from date of randomisation).
N_8 = number of patients with at least eight years of follow-up (from date of randomisation).

N_{10} = number of patients with at least ten years of follow-up (from date of randomisation).

N_1 = number of patients seen within previous 58 weeks (1 y plus 6 week window) of Reference Date
N_f = include if $N_{5/8/10}$ or N_1, the number of patients with adequate follow-up
D_{all} = number of patients randomised (3451)
D_{cens} = number of patients dead or withdrawn before 5 years

The median follow-up will be determined on the population: D_{all} and (D_{all} – D_{cens})

Survival without local recurrence – the number alive without local recurrence (as 1 above) (censored=last date known to be alive or withdrawn)

Local recurrence in the conserved breast: Histologically or cytologically proven recurrence of tumour in the index breast. (censored=last date known to be alive, withdrawn, or dead)

Index quadrant recurrence: Local recurrence as in 1 above in the index quadrant. (censored=last date known to be alive, withdrawn, dead, other quadrant recurrence)

Scar recurrence: Local recurrence as in 1 above within 1cm of the scar. (censored=last date known to be alive, withdrawn, dead, non-scar recurrence)

Other quadrant recurrence: Local recurrence as in 1 above in other quadrants. (censored=last date known to be alive, withdrawn, dead, index quadrant recurrences)

Invasive local recurrence in the conserved breast: As in 1 above, excluding those with only in-situ disease. (censored=last date known to be alive, dead, withdrawn)

Survival without invasive local recurrence: As in 2 above, excluding those with in-situ disease (censored=last date known to be alive, withdrawn, any other type of relapse)

Isolated local recurrence in the conserved breast: As in 1 above, excluding those with distant disease within 3 months of local recurrence. (censored=last date known to be alive, dead, withdrawn)

Survival without isolated local recurrence: As in 2 above, excluding those with distant disease within 3 months of local recurrence. (censored=last date known to be alive, withdrawn, any other type of relapse)

Overall survival: The time interval between randomisation and death. (censored=last date known to be alive or withdrawn)

Breast cancer survival: The time interval between randomisation and breast cancer death (censored=last date known to be alive or withdrawn)

Non-Breast-cancer survival: The time interval between randomisation and non-breast-cancer death (also, cardiac and other cancer deaths separately) (censored=last date known to be alive or withdrawn)

Lung cancer and heart disease deaths (if possible as a subset analysis in smokers) and effect of laterality on non-breast cancer deaths [appendix], and correlation with EBRT dose.

Disease free survival: The time interval between randomisation and any type of breast cancer relapse or any death (censored=last date known to be alive, withdrawn)

Toxicity of surgery and radiotherapy as previously described.

Analysis for all outcomes of the original (Lancet 2010) cohort of the first 2232 patients (mature cohort) as well as of the first 1222 patients (earliest cohort) who be expected to have completed a median follow up of 10 years.
The following will be regarded as EXPLORATORY ANALYSES

Regional recurrence: Recurrence in the axilla or supraclavicular fossa

Axillary recurrence with distant disease: Recurrence in axilla concurrent with distant disease, or within 3 months of diagnosis of distant disease, or after diagnosis of distant disease.

To assess if there is adequate follow-up: The ratios of both $N_f / (D_{all} – D_{cens})$ > 95% as well as N_f / D_{all} >95% will be used. If we find that this is impossible after adequate effort, we will have to reconsider the boundary with Trial Steering Committee.

Checks will be made for bias in follow-up by randomised treatment and/or by centre and statistical models will be used to assess the effect of such a bias. Additional analyses, tables and figures may be produced including analyses to assist in the determination of outliers, unusual data from specific centres or subgroups, skewness, kurtosis, etc. at the discretion of the Trial Statistician. Inclusion of such analyses, tables in the final report is optional.
In addition, planned subgroup analyses will be performed for the main end points: local recurrence, local-recurrence-free survival, overall survival, breast cancer survival and non-breast-cancer survival for patients who are PgR positive vs. PgR negative and ER receptor positive vs. ER receptor negative, method of diagnosis (screening vs symptomatic), use of hormone therapy, and other predictive/prognostic factors.

A cox- proportional hazard model will be used for analysis of any other predictive factors including variables such as age, tumour size, grade, HER2 status, axillary nodal involvement, vascular invasion, margin, whether screen detected or not. Influence of factors such as ASTRO and/or ESTRO criteria, timing of 1st surgery/biopsy in terms of menstrual cycle, time of the day of delivery of radiotherapy, height, weight, bra size, surgical technique and size of applicator used for TARGIT, dose prescription used for TARGIT, fractionation regime for EBRT, delay between initial surgery and delivery of TARGIT in the postpathology stratum, centre and country, use of systemic therapy, or other factors will also be explored provided sufficient data is available. Significant factors will be reported with absolute differences, and/or hazard ratios and 95%CI.

We shall also perform all the analysis for the first 1222 and 2322 patients recruited in the trial, referred to as the Earliest and Mature cohorts in the 2013/14 Lancet publication. We shall also perform these analyses for the first n number of patients in the pre-pathology stratum to enable a comparison with other trials of partial breast irradiation, where n matches the number of patients in those trials.

We shall describe completeness of follow-up, mastectomy rates, proportion of patients suspected and investigated to have local recurrence in each randomised arm, treatment of local recurrence, number of uncontrolled local recurrence at the time of death and use of EBRT in presence of high risk factors and the effect of radiation fractionation on toxicity. For completion, we shall analyse the cancer, toxicity, overall survival and non-breast-cancer survival outcomes as per treatment received (those that received only TARGIT, both TARGIT and EBRT and EBRT only), however, the results of these artificially created groups need to be interpreted with the consideration that TARGIT treatment is only recommended within a risk-adapted approach.

11 References

1. The Supreme Court U. Montgomery v Lanarkshire Health Board. In: Kingdom SCoU, editor. *[2015] UKSC 11*. United Kingdom: The Supreme Court, 2015:1-37.
2. Wickberg A, Holmberg L, Adami HO, Magnuson A, Villman K, Liljegren G. Sector resection with or without postoperative radiotherapy for stage I breast cancer: 20-year results of a randomized trial. *Journal of clinical oncology : official journal of the American Society of Clinical Oncology* 2014;32(8):791-7.
3. Wickberg A, Holmberg L, Adami HO, Magnuson A, Villman K, Liljegren G. Reply to a. Levy et Al. *Journal of clinical oncology : official journal of the American Society of Clinical Oncology* 2014;32(29):3340.
4. Vaidya JS, Wenz F, Tobias JS. Trial supports targeted radiotherapy for early breast cancer but protocol still requires 3 weeks of daily therapy. *BMJ evidence-based medicine* 2018;23(1):38-39.

5. Vaidya JS, Bulsara M, Wenz F, Tobias JS, Joseph D, Baum M. Targeted radiotherapy for early breast cancer. *Lancet* 2018;391(10115):26-27.
6. Corica T, Nowak AK, Saunders CM, Bulsara MK, Taylor M, Williams NR, Keshtgar M, Joseph DJ, Vaidya JS. Cosmetic outcome as rated by patients, doctors, nurses and BCCT.core software assessed over 5 years in a subset of patients in the TARGIT-A Trial. *Radiat Oncol* 2018;13(1):68.
7. Vaidya JS. The Systemic Effects of Local Treatments (Surgery and Radiotherapy) of Breast Cancer. In: Retsky M, Demichelli R, editors. *Perioperative Inflammation as Triggering Origin of Metastasis Development*: Nature, Springer, 2017:227-36.
8. Vaidya A, Vaidya P, Both B, Brew-Graves C, Bulsara M, Vaidya JS. Health economics of targeted intraoperative radiotherapy (TARGIT- IORT) for early breast cancer: a cost- effectiveness analysis in the United Kingdom. *BMJ open* 2017;7:e014944.
9. Vaidya JS, Wenz F, Bulsara M, Tobias JS, Joseph DJ, Saunders C, Brew-Graves C, Potyka I, Morris S, Vaidya HJ, Williams NR, Baum M. An international randomised controlled trial to compare TARGeted Intraoperative radioTherapy (TARGIT) with conventional postoperative radiotherapy after breast-conserving surgery for women with early-stage breast cancer (the TARGIT-A trial). *Health technology assessment* 2016;20(73):1-188.
10. Vaidya JS, Bulsara M, Wenz F, Coombs N, Singer J, Ebbs S, Massarut S, Saunders C, Douek M, Williams NR, Joseph D, Tobias JS, Baum M. Reduced Mortality With Partial-Breast Irradiation for Early Breast Cancer: A Meta-Analysis of Randomized Trials. *International journal of radiation oncology, biology, physics* 2016;96(2):259-65.
11. Vaidya JS, Bulsara M, Wenz F, Coombs N, Singer J, Ebbs S, Massarut S, Saunders C, Douek M, Williams N, Joseph D, Tobias JS, Baum M. Reduced Mortality With Partial Breast Irradiation for Early Breast Cancer Reply. *International Journal of Radiation Oncology Biology Physics* 2016;96(3):707-08.
12. Corica T, Nowak AK, Saunders CM, Bulsara M, Taylor M, Vaidya JS, Baum M, Joseph DJ. Cosmesis and Breast-Related Quality of Life Outcomes After Intraoperative Radiation Therapy for Early Breast Cancer: A Substudy of the TARGIT-A Trial. *International journal of radiation oncology, biology, physics* 2016;96(1):55-64.
13. Coombs NJ, Coombs JM, Vaidya UJ, Singer J, Bulsara M, Tobias JS, Wenz F, Joseph DJ, Brown DA, Rainsbury R, Davidson T, Adamson DJA, Massarut S, Morgan D, Potyka I, Corica T, Falzon M, Williams N, Baum M, Vaidya JS. Environmental and social benefits of the targeted intraoperative radiotherapy for breast cancer: data from UK TARGIT-A trial centres and two UK NHS hospitals offering TARGIT IORT. *BMJ open* 2016;6(5).
14. Vaidya JS, Bulsara M, Wenz F, Joseph D, Saunders C, Massarut S, Flyger H, Eiermann W, Alvarado M, Esserman L, Falzon M, Brew-Graves C, Potyka I, Tobias JS, Baum M, Grp TT. Pride, Prejudice, or Science: Attitudes Towards the Results of the TARGIT-A Trial of Targeted

Intraoperative Radiation Therapy for Breast Cancer. *International Journal of Radiation Oncology Biology Physics* 2015;92(3):491-97.
15. Vaidya JS, Wenz F, Bulsara M, Tobias JS, Joseph DJ, Keshtgar M, Flyger HL, Massarut S, Alvarado M, Saunders C, Eiermann W, Metaxas M, Sperk E, Sutterlin M, Brown D, Esserman L, Roncadin M, Thompson A, Dewar JA, Holtveg HM, Pigorsch S, Falzon M, Harris E, Matthews A, Brew-Graves C, Potyka I, Corica T, Williams NR, Baum M, TARGIT-trialists'-group. Risk-adapted targeted intraoperative radiotherapy versus whole-breast radiotherapy for breast cancer: 5-year results for local control and overall survival from the TARGIT-A randomised trial. *Lancet* 2014;383(9917):603-13.
16. Vaidya JS, Wenz F, Bulsara M, Tobias JS, Joseph D, Baum M. Radiotherapy for breast cancer, the TARGIT-A trial - Authors' reply. *Lancet* 2014;383(9930):1719-20.
17. Wenz FK, Vaidya JS, Bulsara M, Suetterlin M, Sperk E, Ataseven B, Pigorsch S, Feyer PC, Blohmer JU, Kaufmann M, Roedel C, Friese K, Belka C, Solomayer E, Fleckenstein J, Park-Simon T-W, Bremer M, Joseph DJ, Tobias JS, Baum M. TARGIT-A trial: Updated results for local recurrence and survival for the German centers. *Journal of Clinical Oncology* 2013;31(15).
18. Vaidya JS, Wenz F, Bulsara M, Massarut S, Tobias J, Williams N, Joseph D, Baum M. Omitting Whole Breast Radiation Therapy did not Increase Axillary Recurrence in the TARGIT-A Trial. *International Journal of Radiation Oncology Biology Physics* 2013;87(2):S7.
19. Vaidya JS, Bulsara M, Wenz F, Massarut S, Joseph D, Tobias J, Williams NR, Baum M. FEWER NON-BREAST CANCER DEATHS IN TARGIT-A TRIAL: SYSTEMIC BENEFIT OF TARGIT OR LACK OF EBRT TOXICITY. *Breast* 2013;22:S97.
20. Vaidya JS, Bulsara M, Wenz F, Massarut S, Joseph D, Tobias J, Williams N, Baum M. The Lower Non-Breast Cancer Mortality With TARGIT in the TARGIT-A Trial Could Be a Systemic Effect of TARGIT on Tumor Microenvironment. *International Journal of Radiation Oncology Biology Physics* 2013;87(2):S240.
21. Vaidya JS, Bulsara M, Wenz F, Massarut M, Joseph D, Tobias JS, Williams N, Baum M. Fewer non-breast cancer deaths in the TARGIT-A trial Systemic benefit of TARGIT or lack of EBRT toxicity? *Breast* 2013;22(Suppl 1):S97(P235).
22. Vaidya JS, Bulsara M, Wenz F. Ischemic heart disease after breast cancer radiotherapy. *New England Journal of Medicine* 2013;368(26):2526-27.
23. Baum M, Vaidya JS, Bulsara M, Wenz F, Tobias JS, Eiermann W, Joseph D. INSIGHTS INTO THE NATURAL HISTORY OF SUBCLINICAL BREAST CANCER: A BIOLOGICAL FALL OUT FROM THE TARGIT-A TRIAL. *Annals of Oncology* 2012;23(Suppl. 9):ixe 1-ixe 30.
24. Vaidya JS, Baum M, Tobias JS, Wenz F, Massarut S, Keshtgar M, Hilaris B, Saunders C, Williams NR, Brew-Graves C, Corica T, Roncadin M, Kraus-Tiefenbacher U, Sutterlin M, Bulsara M, Joseph D. Long-term results of targeted intraoperative radiotherapy (TARGIT) boost during breast-

conserving surgery. *International journal of radiation oncology, biology, physics* 2011;81(4):1091-7.

25. Vaidya JS, Joseph DJ, Tobias JS, Bulsara M, Wenz F, Saunders C, Alvarado M, Flyger HL, Massarut S, Eiermann W, Keshtgar M, Dewar J, Kraus-Tiefenbacher U, Sutterlin M, Esserman L, Holtveg HM, Roncadin M, Pigorsch S, Metaxas M, Falzon M, Matthews A, Corica T, Williams NR, Baum M. Targeted intraoperative radiotherapy versus whole breast radiotherapy for breast cancer (TARGIT-A trial): an international, prospective, randomised, non-inferiority phase 3 trial. *The Lancet* 2010;376(9735):91-102.
26. Zhao L, Tian L, Uno H, Solomon SD, Pfeffer MA, Schindler JS, Wei LJ. Utilizing the integrated difference of two survival functions to quantify the treatment contrast for designing, monitoring, and analyzing a comparative clinical study. *Clinical trials* 2012;9(5):570-7.
27. Royston P. A combined test for a generalized treatment effect in clinical trials with a time-to-event outcome. *Stata J* 2017;17(2):405-21.
28. Wei Y, Royston P, Tierney JF, Parmar MK. Meta-analysis of time-to-event outcomes from randomized trials using restricted mean survival time: application to individual participant data. *Statistics in medicine* 2015;34(21):2881-98.
29. Royston P, Parmar MK. Restricted mean survival time: an alternative to the hazard ratio for the design and analysis of randomized trials with a time-to-event outcome. *BMC Med Res Methodol* 2013;13:152.
30. Royston P, Parmar MK. The use of restricted mean survival time to estimate the treatment effect in randomized clinical trials when the proportional hazards assumption is in doubt. *Statistics in medicine* 2011;30(19):2409-21.

_TARG_eted _I_ntraoperative radio_T_herapy

vs.

Post Operative Radiotherapy

An international randomised controlled trial to compare targeted intra-operative radiotherapy with conventional post-operative radiotherapy after conservative breast surgery for women with early stage breast cancer

Protocol Version 5.1
July 2010

TARGiT

Intraoperative Radiation Therapy

[Blank page]

Trial Summary

TARGIT is an international randomised clinical trial designed to test the hypothesis that the strategy of delivering a single dose of targeted intraoperative radiotherapy (IORT) in patients eligible for breast conserving therapy (with the addition of whole breast radiotherapy in those patients at high risk of recurrence elsewhere in the breast [e.g. lobular carcinomas and extensive intraductal component]) is equivalent to a conventional course of post-operative external beam radiotherapy (EBRT). The primary endpoints are local and loco-regional recurrence rates.

It is a pragmatic trial in which each participating centre has the option to define more restrictive entry criteria than in the core protocol. Only centres with access to the Intrabeam® (Carl Zeiss) enter patients into the trial.

Eligible patients are those with tumours of good prognosis suitable for breast conserving surgery. After giving consent patients are randomised to either IORT or to EBRT. They may receive any other adjuvant treatments as deemed necessary, except for neoadjuvant therapy. The protocol requires that patients be followed at six monthly intervals for five years and then annually.

Any enquiries about the trial should be addressed to the International Steering Committee via the TARGIT Trial Operations Office:

TARGIT Trial Operations Office
Clinical Trials Group
Division of Surgery and Interventional Science
UCL Medical School
Centre for Clinical Science and Technology
Clerkenwell Building, Whittington Campus
Highgate Hill
London N19 5LW, UK

Tel +44 (0) 20 7288 3970
Fax +44 (0) 20 7288 3969

Email targit@ctg.ucl.ac.uk

Website www.targittrial.org and www.targit.org.uk

Trial Sponsor University College London (UCL)
ISRCTN 34086741
ClinicalTrials.gov Identifier NCT00983684

International Steering Committee

**Co-chairmen**	**Michael Baum**, Emeritus Professor of Surgery, University College London, UK
	**David J Joseph**, Professor of Radiation Oncology, Sir Charles Gairdner Hospital, Perth, Australia
**Statistician**	**Max Bulsara,** University of Notre Dame, Fremantle, Australia
**Members**	
**UK**	**Jeffrey S Tobias,** University College London Hospitals, London
	**Jayant S Vaidya**, Whittington Hospital, London
	**Mohammed Keshtgar**, Royal Free Hospital, London
	**John Dewar**, Ninewells Hospital, Dundee
	**Mary Falzon**, University College Hospital, London
	**April Matthews**, Patient Advocate, London
	**Norman Williams** Clinical Trials Group, UCL, London
**Australia**	**Christobel Saunders,** Sir Charles Gairdner Hospital, Perth
	**Tammy Corica,** Sir Charles Gairdner Hospital, Perth
**Italy**	**Samuele Massarut**, Centro di Riferimento Oncologico (CRO), Aviano
**USA**	**Laura Esserman,** University of California San Francisco
	**Michael Alvarado,** University of California San Francisco
**Germany**	**Frederik Wenz,** University Medical Centre Mannheim
	**Wolfgang Eiermann,** Rotkreuzklinikum Frauenklinik, München
	**Marc Sütterlin**, University Medical Centre Mannheim
	**Uta Kraus-Tiefenbacher,** University Medical Centre Mannheim
**Denmark**	**Henrik Flyger**, Copenhagen
**Carl Zeiss**	**Dietrich Wolf,** Carl Zeiss, Germany
	**Karl Weissbach**, Carl Zeiss, Germany
	**Matthias Benker,** Carl Zeiss, Germany
	**Jeff Rospert**, Carl Zeiss, USA

Signature Page

TARGIT

An international randomised controlled trial to compare targeted intra-operative radiotherapy with conventional post-operative radiotherapy after conservative breast surgery for women with early stage breast cancer.

Version 5.1

International Steering Committee Co-Chairmen

I have read the trial protocol and will ensure that the trial is conducted according to the terms of that protocol and in accordance with agreed international standards for randomised clinical trials.

Professor Michael Baum
Emeritus Professor of Surgery
Clinical Trials Group
Division of Surgery and Interventional Sciences
UCL Medical School
Centre for Clinical Science and Technology
Clerkenwell Building, Whittington Campus
Highgate Hill
London N19 5LW, UK

Signature………………………………………………… Date ……………………..

Professor David Joseph
Professor of Radiation Oncology
Department of Radiation Oncology
Sir Charles Gairdner Hospital
Nedlands 6009
Western Australia

Signature………………………………………………… Date ……………………..

[Blank page]

Contents

Trial Summary 3
International Steering Committee 3
Signature Page 5

BACKGROUND 9
1.1 The Targeted Intra-operative Radiotherapy (TARGIT) Technique 10

OBJECTIVES 12
2.1 Main Objective 12
2.2 Secondary Objectives 12

TRIAL DESIGN 13
3.1 Core Protocol 13
3.1 Trial Schema - Stratum 1 - Pre Pathology Trial Entry 15
3.2 Trial Schema – Stratum 2 - Post Pathology Trial Entry 16
3.3 Contralateral Cancer Trial Entry 16

END POINTS 17

SELECTION AND ENTRY OF PATIENTS 18
5.1 Eligible patients 18
5.2 Exclusion criteria 18
5.3 Patient entry to the trial 19
5.3.1 Stratum 1 - Pre Pathology Entry 19
5.3.2 Stratum 2 - Post Pathology Entry 19
5.3.3 Stratum 3 - Contralateral Entry 19
5.3.4 Patient Consent 19

TREATMENTS 20
6.1 Treatment Policy Statements 20
6.2 Surgery 20
6.3 Pathological Examination 22
6.4 Radiotherapy 22
6.5 Adjuvant Systemic Therapy 24

PATIENT FOLLOW-UP AND RECORDING OF EVENTS 25
7.1 Follow-up and notification of recurrence, adverse events and death.........25
7.2 Recording of recurrence/new malignancies...25
7.3 Guidelines for the Management of Recurrence...25
7.4 Adverse Events 26
7.5 Deaths 27
7.6 Withdrawal of Consent..27

DATA OWNERSHIP AND PUBLICATION..28
8.1 Trial Administration 28

STATISTICAL CONSIDERATIONS...29
9.1 Patient Numbers and Power Calculations 29
9.2 Revision of Patient Numbers 29
9.3 Early Stopping 30
9.4 Statistical Analysis...30
9.5 Additional Sub-protocols 30

ETHICAL CONSIDERATIONS 31
10.1 Data Monitoring Committee (DMC) 31

QUALITY ASSURANCE...32

REFERENCES 33

APPENDICES

1. **The papers related to the original pilot study (1a, 1b and 1c)**
2. **BASO Guidelines are at** http://www.baso.org.uk/Downloads/BASOguidelines_2005.pdf
3. **Blank Treatment Policy Document**
4. **Examples* of Subject information sheet + consent form (stratum 1, single procedure)**
5. **Examples* of Subject information sheet +consent form (stratum 2, subsequent procedure)**
6. **Guidelines for Conventional Breast Irradiation**
7. **Dose prescription for intraoperative radiotherapy**
8. **Targit Brochure (example)**

*UK example: Other examples are available as separate documents

SECTION 1

BACKGROUND

Over the past 30 years, there has been a dramatic change in the local management of breast cancer – the very radical operations being replaced by more conservative surgical procedures, with the widespread use of radiotherapy in conjunction with the wide local excision of the tumour. This shift away from radical surgery has been prompted by results from randomised clinical trials that have clearly demonstrated that breast conserving surgery followed by radiotherapy is equivalent to more radical procedures in terms of local control and overall survival[1-3]. However, although the operation is 'conservative' the intention is 'radical' with the radiotherapy fields encompassing virtually all of the tissues previously excised by radical mastectomy. We propose that this approach be changed and have already rehearsed the biological and clinico-pathological arguments for avoiding unnecessary treatment to the whole breast[4;5].

In part, these are based on the fact that in large studies of breast conserving therapy more than 90% of early breast recurrences have been found to occur at the site of the original primary tumour site. This is true whether or not radiotherapy is given[6] and whether or not the margins are involved[4;7]. Furthermore, when detailed examination of mastectomy specimens are performed using radiological-histological correlational methods[4], small additional invasive or in situ cancer foci are found in over 60% of patients, with 80% of these situated remote from the index quadrant. The relative distribution of primary tumour and these occult foci in the four breast quadrants is significantly different. Hence it appears that these occult cancer foci do not in general give rise to local recurrence, which more probably develops from the cells that surround the primary tumour. These may be overtly malignant or morphologically normal, yet capable of malignant progression, as evident by the loss of heterozygosity in these 'normal' cells within the index quadrant[8;9].

We feel that the next step is a clinical trial to test whether radiotherapy to the index quadrant alone can achieve as good a local control as radiotherapy to the whole breast[10-15]. This approach of irradiating the index quadrant alone has been tested in two clinical trials and in fact (contrary to popular myth) the results of these trials are encouraging. The Christie Hospital Trial[16;17] randomised 708 patients to receive either the standard wide field radiotherapy or a limited field to the index quadrant. Overall there was a higher recurrence rate in the latter arm. However, when the results were analysed according to the type of the primary tumour, it was found that limited field radiotherapy was only inadequate in patients with infiltrating lobular cancers or cancers with extensive intraductal component (EIC). In the 504 cases of infiltrating duct carcinoma, there was no significant difference in the local recurrence rates between the two arms. In a much smaller (n=28) study [18] a single continuous application of an iridium-192 implant delivering 55 Gy over 5-6 days was compared to standard radiotherapy including the whole breast plus boost to the tumour bed. The authors found a 20% increase in local recurrence rates in the patients who had received only an implant. However, as discussed in a letter in response to the study[19], the Biologically Effective Dose (BED) of the implant-only arm was 20% lower than the conventional radiotherapy arm and these patients were a high risk group (12 patients were node positive and 15 had involved margins) and this could explain the difference.

1.1 The Targeted Intra-operative Radiotherapy (TARGIT) Technique

The recent development of a new method has made possible the delivery of radiation directly to the tissues at the site of the primary tumour. The Intrabeam® (originally developed in collaboration with the Photoelectron Corporation in Massachusetts, USA by now manufactured by Carl Zeiss, Germany) is a simple but ingenious device, in essence a miniature electron beam-driven X-ray source which provides a point source of low energy X-rays (50kV maximum) at the tip of a 3.2mm diameter tube. The radiation source can be inserted into the area of interest immediately after excision of the tumour (Figure 1) and switched on for 20-35 minutes to provide intra-operative radiotherapy accurately targeted to the tissues that are at highest risk of local recurrence. The physics, dosimetry and early clinical applications of this soft x-ray device have been well studied and the probe has already been used for treatment of human malignant brain tumours.[20;21]

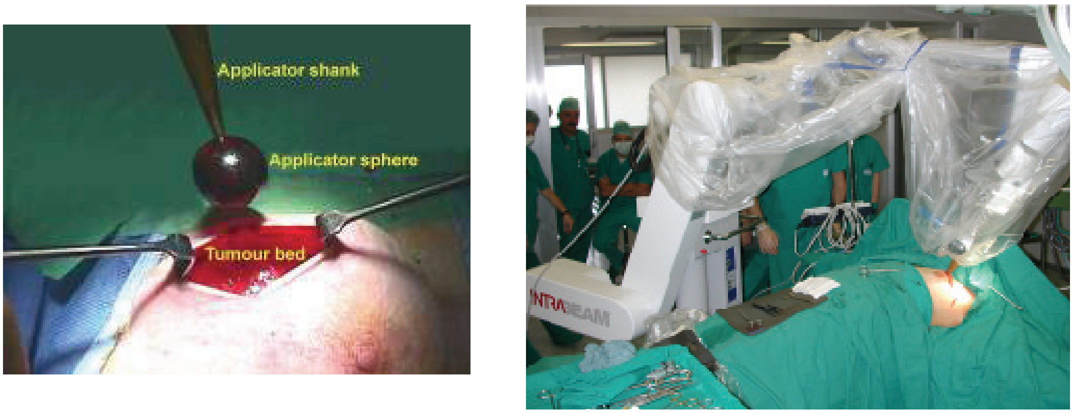

Figures 1 (a) and (b)

Figure 1 (a): The applicator being placed in the tumour bed, immediately after the excision of the tumour.

Figure 1 (b): The x-ray source is delivered to the tumour bed utilizing a surgical support stand. The sterile applicator is joined with a sterile drape that is used to cover the stand during treatment delivery.

It was developed for use for breast cancer and was first piloted at University College London[22-24]. The device has subsequently received FDA approval for use in any part of the body. For use in the breast, the radiation source is surrounded by a spherical applicator (see Figures 1 (a) and 2). The spherical applicator is specially designed to produce a uniform field of radiation at its surface, enabling delivery of an accurately calculated dose to a prescribed depth. It is inserted in the tumour bed and apposed to it with surgical sutures and/or other means. Since the radiation consists of soft X-rays, the beam rapidly attenuates reducing the dose to more distant tissue. Full measurements and calibration are carried out in a water phantom and/or in materials that simulate the radiation absorption properties of the breast. Depending upon the size of the surgical cavity, various sizes of applicator spheres are available. The radiation received is proportional to the time the machine is switched on. The precise dose rate depends on the diameter of the applicator and the energy of the beam, both of which may be varied to optimise the radiation treatment. The radiation doses at various distances from the cavity margin are shown for the simulated assembly in Appendix. For example, a dose of about 20 Gy can be delivered in about 20 minutes to the tumour bed apposing the applicator. Following the treatment delivery, the radiation is switched off, the applicator removed from the wound, and the wound closed in the normal way.

The x-ray source is small and lightweight (weight =1.8 Kg; dimensions: X-ray generator body 7 x 11 x 14 cm; applicator: 16 cm long conical applicator shank with a 2 to 5 cm sphere

at the tip) and is mounted on the surgical stand and balanced for ease of delivery and support during treatment. If necessary, the chest wall and skin can be protected (>93% shielding) by radio-opaque tungsten-filled silicone shields which can be cut to size on the operation table, another advantage of using soft x-rays. With this elegant approach the pliable breast tissue around the cavity of surgical excision wraps around the radiotherapy source, i.e. the target is 'conformed' to the source. This simple, effective technique avoids the unnecessarily complex and sophisticated techniques of using interstitial implantation of radioactive wires to provide high dose radiotherapy to the tumour bed or the even more complex techniques necessary for conformal radiotherapy by external beams from a linear accelerator. The quick attenuation of the radiation dose allows the treatment to be carried out in the routine theatre. The walls usually incorporate shielding for microwave radiation from electronic equipment such as mobile phones and as such provide enough protection to the staff. Furthermore, the dose attenuates rapidly, so that the highest radiation dose is received by tissue nearest the primary tumour and a much lower dose at the skin. Thus in theory, the biological effect and cosmetic outcome could be improved.

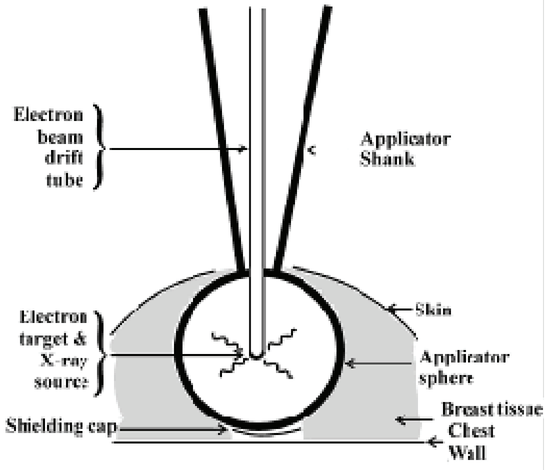

Figure 2 The Intrabeam System (PRS). The electrons are generated and accelerated in the main unit and travel via the electron beam drift tube which is surrounded by the conical applicator sheath such that its tip lies at the epicentre of the applicator sphere. Once the electrons hit the inner surface of the hemisphere at the tip, x-rays are generated. Thus a uniform radiation dose rate is available at the surface of the applicator sphere. (taken from Vaidya et al, Ann Oncol 2001)

Twenty-five cases have been treated in a pilot study at UCL using IORT as a peri-operative replacement for the boost dose of radiotherapy; full dose external beam treatment was subsequently given[22]. An updated series of 302 cancers in 301 patients has been recently published[25] along with favourable toxicity and cosmetic outcome results of individual cohorts [15;26-28]. A mathematical model of TARGIT developed recently (funded by Cancer Research UK) suggests that it could be superior to conventional radiotherapy [29;30]

In summary, this technique thus offers many advantages but needs full evaluation in a randomised trial, which is now described in detail in the following protocol.

SECTION 2

OBJECTIVES

This is a pragmatic trial that compares two treatment policies in patients with early breast cancer who have undergone local excision of a good prognosis tumour. The conventional policy is that each patient receives a radical course of external beam radiotherapy (with or without a boost) according to local treatment guidelines. The experimental policy is to give targeted intra-operative radiotherapy in a single dose recognising that some patients randomised to this treatment, because of unfavourable features found subsequently in the pathological examination of the excised lesion, will need to have external beam therapy in addition (without the boost which has been provided by the targeted dose). This could happen in 10 to 15% of cases. A nested study allows randomisation of patients to IORT or EBRT after the pathological examination of the removed lesion. Patients in this randomisation who are allocated IORT will require a second surgical procedure for administration of the radiation.

The objective of the trial, regardless of the exact procedure followed, is to provide local tumour control but the two treatments involve very different time and financial commitments on behalf of the individual patient and the health service.

2.1 Main Objective

To evaluate whether a single fraction of radiotherapy given intra-operatively and targeted to the tissues at the highest risk of local recurrence is equivalent to standard post-operative radiotherapy after breast conserving surgery in women with early stage breast cancer in terms of local relapse within the treated breast.

2.2 Secondary Objectives

To compare the treatment arms with respect to:

- Site of relapse within the breast
- Relapse-free survival and overall survival
- Local toxicity/morbidity

Separate protocols will address other endpoints:

- Cosmetic outcome
- Patient satisfaction and quality of life
- Health economics and cost-benefit
- Patient Preference

SECTION 3

TRIAL DESIGN

3.1 Core Protocol

A pragmatic randomised trial has been designed to directly compare the outcome, primarily in terms of local control, of two approaches of adjuvant radiotherapy. Nested within the pragmatic trial is a more selective approach that may be employed in some centres in some or all patients (for example, the Australasian protocol uses more selective eligibility criteria and is available on request from the trial centre).

Patients selected for breast-conserving surgery who are considered to have a low risk of local recurrence are eligible for the trial once they have received information and given their consent.

This document describes the 'Core Protocol' for the International TARGIT trial. However, individual centres may describe tighter entry and management criteria than given here. These are to be documented in advance (see Section 6.1 and Appendix) and all trial patients treated according to this pre-declared policy. All patients randomised within this protocol will be analysed under an 'intention to treat policy' (ITT) but stratified analyses according to the randomisation strata will also be performed (see below). In addition the group randomised under the tighter criteria (as for example in the Australasian Protocol) will be analysed both in the overall ITT analysis and as a separate stratum at a later date when sufficient events are available.

Randomisation will be stratified firstly according to participating site. Then patients will be randomised into the trial in one of three strata; the rationale for each is given below. Investigators may determine in advance which strata they wish to use for entry of patients from their centre and stipulate this in the Treatment Policy Statement. If a centre has agreed entry to all three strata the decision about which strata needs to be made prior to randomisation, for each individual.

Stratum 1 – Pre Pathology Entry

This stratum addresses the broad policy issue of using IORT. It allows entry of patients who have been diagnosed with 'early' breast cancer and are suitable for treating conservatively (small tumour and no gross nodal involvement). Preferably, tumours should not be more than 3.5 cm in size, as the largest applicator size is 5cm. Patient consent is sought and randomisation is carried out prior to the surgical removal of the tumour. Postoperatively, some characteristics may be found that militate against a single intraoperative dose of radiotherapy (e.g. lobular carcinoma, grade III histology, positive margins at first excision, lymphovascular invasion, involved axillary nodes etc). In these cases, (as per the centre's treatment policy) the patient will be recommended to have a full course of external beam radiotherapy. The intraoperatively delivered radiotherapy replaces the boost in this instance. Some of these adverse characteristics could be known preoperatively with a core biopsy which is now generally encouraged. The two arms are compared on the basis of the policy to provide local tumour control, accepting that some of the patients in the IORT arm will also have EBRT. This stratum is important because it tests the 'real world' policy; if at the end of the trial the IORT patients are found to have achieved local control equivalent to EBRT and IORT using the Intrabeam becomes more widely practised it is the most likely way it will be implemented.

The trial organisers are well aware that there is a potential bias in this stratum. The surgeon performing the local excision and placing the applicator in the wound will be aware that the patient is allocated to the IORT arm. Although the surgical excision is supposed to be similar in both arms, the surgeon may, consciously or unconsciously, remove a wider margin of normal tissue, or conversely, with the fore-knowledge of imminent accurately delivered radiotherapy, pay less attention to excision margins. The documentation requests the size and weight of the surgical specimen as well as margin status so that any differences between the groups will be known. The alternative of performing 'sham' IORT is not practical or reasonable because of several reasons including ethical, clinical reasons and theatre time. However the most important point is that in this trial, two policies are being compared, not the procedures. Even if at the end of the day, patients randomised to IORT in this stratum do experience slightly larger surgical resections this is considered part of the policy of giving IORT and is likely to be continued if the practise is continued outside the trial. The patients will be asked if they find the cosmetic outcome of the procedure acceptable and this will be also assessed objectively. Ultimately, the effects of the two strategies will be analysed taking both the local recurrence, cosmetic outcome and the convenience and cost saving all together.

Stratum 2 – Post Pathology Entry

Patients will be randomised for entry to the trial only once the pathological characteristics of the tumour have been reported. These patients will have local surgery as per usual practice and if the tumour fits within the criteria defined in the protocol and the investigator's Treatment Policy she will be asked for consent to enter the trial and if given and allocated to the IORT arm she will have further surgery at which the wound is opened, the probe inserted and the dose given.

This stratum of patients addresses a much more explanatory question as their entry to the trial will be strictly defined on pathological grounds. However, the disadvantage for these patients is that they must have a second surgery. This is unlikely to be the way the Intrabeam is used in the majority of cases should the trial show that good local control is provided. Including these patients will allow an assessment of the local control obtained in very good prognosis patients.

This strategy also allows for the entry of patients who have already received surgery at an outlying centre.

Stratum 3 – Entry Following Diagnosis of a Contralateral Breast Cancer

Most patients who require treatment for a metachronous breast cancer are excluded from entry to trials of local treatment. Although there are good reasons for this, later development of recurrence cannot be ascribed to the first or second breast tumour for instance, they do need to be treated and intraoperative treatment may be particularly suitable. The trial will only assess this stratum for ipsilateral local control and their data will be censored for other endpoint assessments.

3.1 Trial Schema - Stratum 1 - Pre Pathology Trial Entry

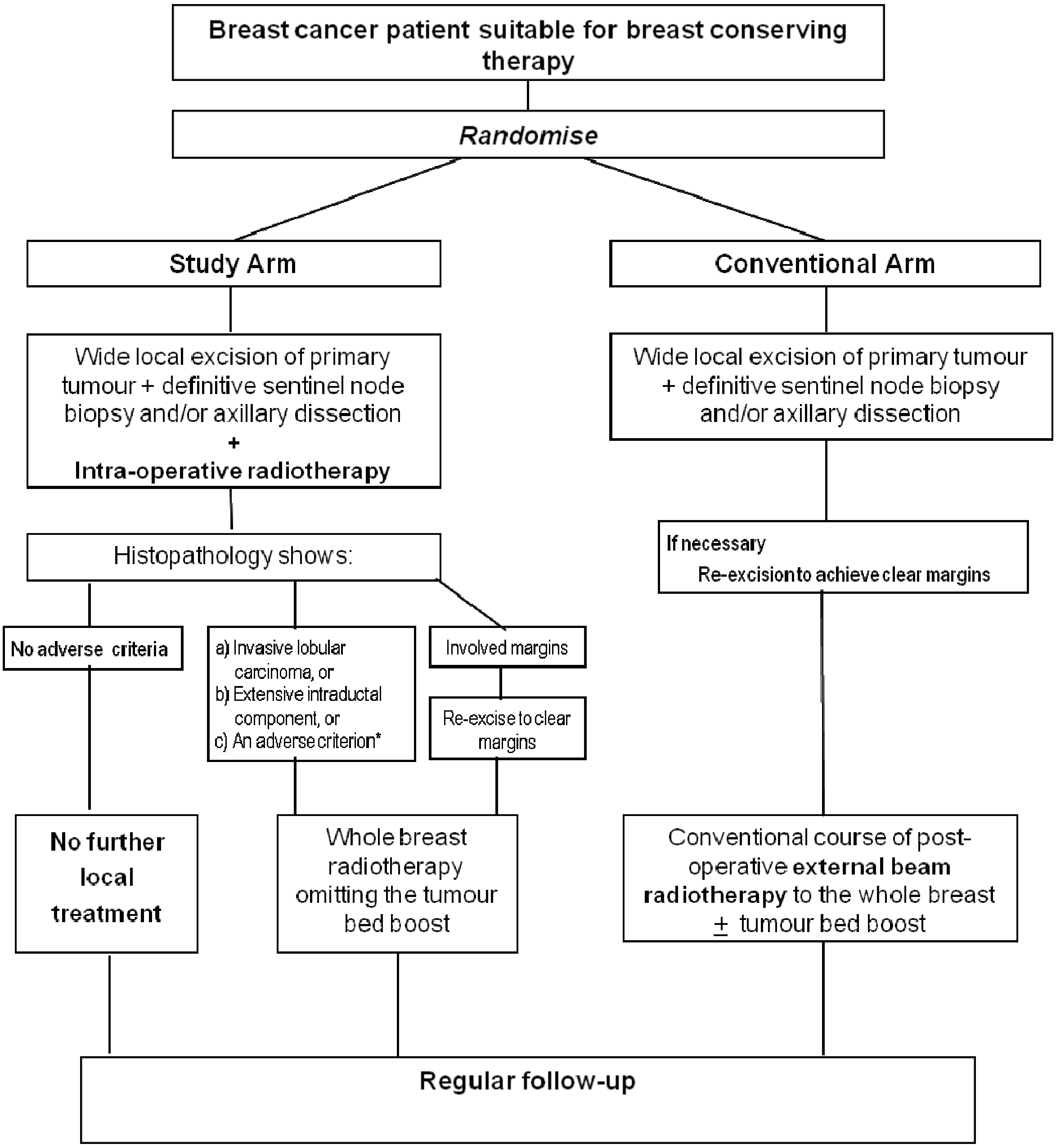

*Decided by each centre at the outset - such as grade III, node involvement, lymphovascular invasion, etc.
Adjuvant systemic therapy should be delivered as and when appropriate

3.2 Trial Schema – Stratum 2 - Post Pathology Trial Entry

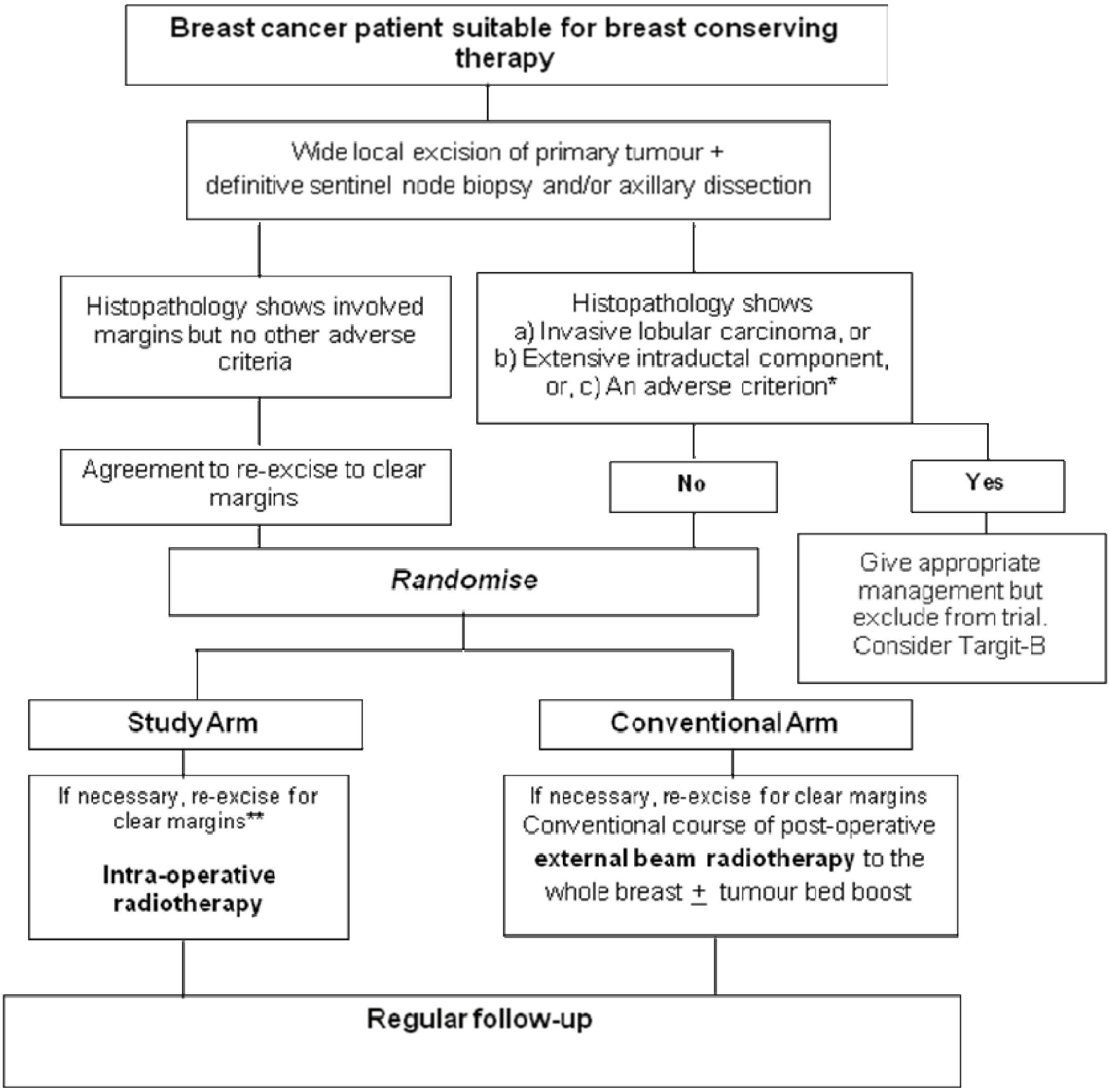

*Decided by each centre at the outset - such as grade III, node involvement, lymphovascular invasion, etc
**If re-excision does not achieve clear margins and IORT has already been delivered, then further re-excision to clear margins should be performed followed by EBRT (without boost).
Adjuvant systemic therapy should be delivered as and when appropriate

3.3 Contralateral Cancer Trial Entry

These patients are randomised in a separate stratum but are managed according to either of the strategies shown in the above two schemas.

SECTION 4

END POINTS

Local tumour control (defined as no recurrent tumour in the ipsilateral breast)
Patients will be regularly monitored as per the individual centre's policy provided this meets the minimum criteria for follow-up of symptomatic breast cancer patients as defined by the Breast Specialty Group of the British Association of Surgical Oncology (Appendix).

Confirmation of recurrence will follow clinical examination and cytology or biopsy. See Section 7.

Site of relapse within the breast will be recorded in order to assess whether the recurrence is at the site of the initial tumour or at a new site and whether it has occurred within the treated field (IORT). The trial CRFs will detail how to record the site of the initial tumour and any subsequent breast recurrence.

Relapse-free survival will be recorded as the time interval between randomisation and the date of confirmation of recurrence. The actual date to be used is the clinic day on which the investigations that led to a confirmed diagnosis of the recurrence were requested. Relapse-free survival will include any recurrence of breast cancer or death without a prior report of relapse.

Overall survival will be the time interval between randomisation and death.

Local toxicity and morbidity will be recorded as adverse events related to the primary treatment of the breast cancer. The expected toxicities of acute skin reaction, wound infection, wound breakdown, late skin reactions (i.e. after 90 days) and pain due to radiation will be graded according to RTOG, LENT SOMA or CTC criteria. (See trial CRFs for more details). All other toxicities will be recorded and graded according to standard clinical criteria.

Cosmesis will be assessed objectively (using photographs) by a physician and a breast care nurse not participating in the trial will be performed including assessment of wound fibrosis/ scar texture at 2 years and at 5 years. The assessors will be kept blinded as to which of the treatments each patient received. Photographs will be assessed for cosmetic outcome and normal tissue damage using a standardised rating scale. (Details will be provided in a sub-protocol.)

Patient satisfaction will be determined from patient views about delivery of treatment and the acceptability of the cosmetic result and will be elicited at 6 weeks and at 2-3 months (for those not receiving chemotherapy) or at 8-9 months (for those receiving chemotherapy) and at similar times after the completion of postoperative radiotherapy for those in the control arm. (Details will be provided in a sub-protocol).

Health economics will be assessed in a sub-protocol to evaluate the cost of the new treatment in comparison to standard breast irradiation will be developed. (Details will be provided in a sub-protocol).

Patient preference has already been piloted in Australia. This investigates the level of inferiority that a patient is ready to accept for the convenience of single-dose radiotherapy (of course assuming that IORT is potentially inferior, when in reality it could be superior). The protocol developed as a result of this pilot will be used in participating centres around the world to assess this and guide ultimate implementation of trial results in the real world.

SECTION 5

SELECTION AND ENTRY OF PATIENTS

5.1 Eligible patients

All patients, aged 45 years or older, with operable <u>invasive</u> breast cancer (T1 and small T2, N0-1, M0), confirmed by cytological or histological examination, who are suitable for breast conserving surgery are eligible. Those with previously diagnosed and treated contralateral breast cancer may be entered but will be randomised to a separate stratum. Individual centres may wish to restrict entry to a more exactly defined subset of patients, in which case, only patients with these characteristics may be entered by that particular centre. For example, centres may decide at outset to recruit only women over 50 or even over 65 years of age. Such policies must be pre-defined in writing and sent to the International Steering Committee. (See Section 6.1 and Appendix 3).

Before entering any patient into the trial the local investigator should check that the patient would be available for regular follow-up (according to local policies) for at least ten years.

5.2 Exclusion criteria

- More than one obvious cancer in the same breast as diagnosed by clinical examination, mammography or ultrasonography.
- Bilateral breast cancer at the time of diagnosis.
- Ipsilateral breast had a previous cancer and/or irradiation.
- Patients *known* to have BRCA2 gene mutations, but testing for gene mutations is *not* required
- Lobular cancer or Extensive intraductal Component (EIC =>25% of the tumour is intraductal) on core biopsy or initial pathology (if performed)
- Patients undergoing primary medical treatment (hormones or chemotherapy) as initial treatment with neoadjuvant intent of reducing tumour size should be excluded; those given short duration (up to 4 weeks) systemic therapy can be included.
- Patients presenting with gross nodal disease, considered to be clinically malignant or proven cytologically or by scanning. In general, 4 or more positive nodes or extranodal spread are not suitable for Targit alone and should receive EBRT as well. However, individual centres may decide that anything more than micrometastasis should receive EBRT.
- Patients with any severe concomitant disease that may limit their life expectancy.
- Previous history of malignant disease *does not* preclude entry if the expectation of relapse-free survival at 10 years is 90% or greater.
- Any factor included as exclusion criterion in the local centre's Treatment Policy. This is particularly relevant to patients entered into the post pathology stratum.
- No more than 30 days can have elapsed between last breast cancer surgery (not axillary) and entry into the trial for patients in the post-pathology stratification.

5.3 Patient entry to the trial

Patients will be entered to the trial only after being given full information about the trial including a written information sheet (see examples in Appendix). Each patient should be given time to consider participation and ask questions before giving consent by signing the Patient Consent Form (see example see Appendix). A patient deemed suitable for the trial will only be randomised once informed consent has been freely given.

5.3.1 Stratum 1 - Pre Pathology Entry

Those patients who are planned to have IORT at the time of primary surgery will be randomised in this stratum after giving consent but prior to the planned surgery.

5.3.2 Stratum 2 - Post Pathology Entry

These patients may be informed of the trial prior to surgery but should only be asked to give consent once the wide local excision has been performed and full histological examination of the tumour has shown that the patient is eligible. Re-excision to clear margins may be performed if required, provided no other adverse histopathological features were found. If these patients are randomised to, and indeed receive IORT and it is later found that the re-excision is again involved, then further re-excision should be performed until clear margins are obtained, and the patient given whole breast EBRT as well, without the tumour bed boost.

5.3.3 Stratum 3 - Contralateral Entry

If the patient has a previous contralateral breast cancer, then they will be entered as a separate stratum irrespective of whether IORT is being planned as a single or second procedure. The numbers are expected to be too small to split this stratum further.

5.3.4 Patient Consent

The example Patient Information Leaflets and Consent Forms may be modified to meet local criteria but must be approved by the appropriate ethics committee/IRB.

Included on the generic consent form is the optional opportunity for the patient to make a gift of the tissue blocks of the tumour for possible translational investigations. Any tissue donated in this way should only be used with the approval of the International Steering Committee.

SECTION 6

TREATMENTS

6.1 Treatment Policy Statements

Prior to entry of any patients each centre will register with the International Steering Committee and complete a Policy Statement (Appendix 3), which will define the categories of patients to be entered (e.g. patients over 50 years, N0) together with some details of treatment policy (e.g. fractionation and dose of conventional radiotherapy to be used). Any change to practice during the course of the trial must be notified to the Steering Committee in writing, prior to implementation. This is to enable the Steering Committee to audit the patients entered and to confirm that treatment remains true to the core protocol.

Only clinical centres with the Intrabeam® or those who are able to refer patients to such a centre may enter the trial. Centres with newly acquired equipment must consult the TARGIT Trial Operations Centre prior to entering patients into the trial. A confirmation of the quality control of the system set up must be received at the Operations Centre before randomisation can begin. (See also Section 6.4.2)

6.2 Surgery

All patients will have local excision of the primary tumour following appropriate clinical work-up. No special assessments prior to randomisation will be required although mammography and ultrasound are recommended to try to exclude multi-focal disease and to determine as accurately as possible the size of the tumour.

Surgery will be according to usual local practice. Complete macroscopic excision of the tumour is required. The aim of the local excision should be to achieve the widest margin of excision whilst maintaining a good cosmetic outcome (aim for margins of >10mm). The final histological margin should be ≥ 1mm clear of all invasive and in-situ disease.

For superficial tumours an ellipse of overlying skin should be excised. The depth of resection will depend on the position of the tumour within the breast and the size of the breast but in most instances will extend to the pectoral fascia.

In all patients, but especially in those women with impalpable tumours where pre-operative localisation has been performed, the specimen should be well orientated with sutures or clips according to local protocols and x-rayed intra-operatively. The specimen x-ray should be examined in theatre to ensure complete excision of the lesion and to help with the assessment of adequacy of the margins. Further tissue should be taken (and marked) from a margin if the x-ray abnormality extends near the margin.

Either a standard sentinel node biopsy or at least level II axillary node clearance must be performed in all patients. Similar surgical techniques must be employed in all patients regardless of the randomisation. Wound closure must be performed meticulously (air and water-tight) as described [23;24] and sutures (if non-absorbable), should remain in place for 14 days.

6.2.1 Pre Pathology Stratum

6.2.1.1 Patients with positive margins

If final pathology (Section 6.3 below) shows involved or close margins (evidence of invasive or in situ tumour at, or within 1mm, of an excision margin) re-excision is strongly recommended. In some cases, this may necessitate a mastectomy.

For patients who have already received IORT, such re-excision to clear margins should be followed by external beam radiotherapy excluding the boost.

6.2.1.2 Patients with markers of poor prognosis (e.g., lobular cancer and Extensive Intraductal Component (EIC))

It is recommended that patients found on pathological examination of the operation specimen to have either invasive lobular cancer or extensive intra-duct component (or other adverse criterion such as grade 3 tumour, lymphovascular invasion, node involvement, etc) as defined by the local centre) receive external beam radiotherapy since these patients are at a higher risk of developing recurrence in the ipsilateral breast at a site other than that of the excised primary. For those patients randomised to intra-operative radiation this will be in addition to the treatment they have already received and it is recommended that any boost is omitted. These patients shall remain in the trial. However, most of such patients (e.g., lobular carcinoma) would have not been included in the first instance if a preoperative core biopsy had been performed; we recommend this.

Alternatively, a mastectomy may be performed if it is deemed necessary, based on the final histopathology, multidisciplinary meeting and joint consultation between the patient and the clinician, irrespective of the arm of randomisation. This must be recorded on the patient's trial case report forms (CRF); the patient should remain in the trial and continued to be followed according to the protocol.

6.2.2 Post Pathology Stratum

Patients randomised in this stratum should have the initial surgery performed and the histopathological examination completed preferably with clear margins confirmed before being randomised. If no other adverse pathological features are present, patients with involved margins may be randomised provided that, repeat excision to clear margins is performed prior to radiotherapy (IORT or EBRT). If after delivering IORT it is found that the re-excision margin is also involved, then these patients should received EBRT as well, as repeated positive margins are a poor prognostic factor.[31]

The procedure for delivering the IORT will be the same as that described in Section 6.4.1 below, including the administration of a prophylactic antibiotic just after the induction of anaesthesia. In this group of patients, special care should be taken to ensure the wound closure is meticulous, air- and water-tight and sutures are not removed for at least 2 weeks. If an absorbable suture is used it should be at least 3-0 in thickness, should not be absorbable within 2 weeks (do not use "rapide") and Steristrips should be used and left in situ for 2 weeks.

6.2.3 Contralateral Stratum

Patients should be managed as appropriate in the pre or post pathology stratum.

6.3 Pathological Examination

Data from pathological examinations should be recorded on the appropriate data collection forms and all reporting of pathological findings are to be in accordance with the TNM Classification. We recommend that the minimum dataset as defined in the British Association of Surgical Oncologists' guidelines should be recorded.

6.4 Radiotherapy

6.4.1 Intra-operative radiotherapy

The technique of the operation and the delivery of radiotherapy have been described in a video available from the TARGIT Trial Operations Office.

Preparation of the Intrabeam

Intra-operative radiotherapy will either be delivered in the operating theatre immediately after the removal of the tumour or as a subsequent procedure, a short time later.

The procedure has been described in detail[22-24] The papers presentations and video related to TARGIT are available at www.targit.org.uk or www.dundee.ac.uk/surgery/targit/ and a video demonstrating the operative technique is at www.theijs.com/media. A brief description is given below:

All patients should receive a prophylactic dose of antibiotic just before skin incision. The device and the arm of the stand are wrapped in a sterile clear plastic cover (see Fig 1). The individual applicators are sterilised prior to the theatre session. The size of the sphere is determined at surgery by the surgeon and/or the radiation oncologist. An appropriately sized Intrabeam sphere fits comfortably without tension in the surrounding tissue so that the skin and subcutaneous tissues can be gathered with a purse string suture over the sphere. Any other apparatus to assist this apposition may also be used.

It is essential that complete haemostasis is achieved before insertion of the applicator sphere, because even a small ooze of blood can distort the cavity around the sphere and significantly change the target dose. The applicator sphere is inserted into the surgical cavity and a deep surgical purse string suture is inserted in the breast tissues to bring together the target breast tissue so that it applies well to the surface of the Intrabeam applicator sphere and holds it in place during treatment. The skin, but not the breast tissue, should be everted and held away from the delivery device by surgical sutures to prevent direct contact with the sphere. One patient in the pilot series described[22] did develop an area of skin necrosis. It is important to keep the skin at a distance of at least 1cm from the applicator.

If necessary, protective caps (made from tungsten impregnated rubber available from Carl Zeiss) may be fashioned by the surgeon to protect deep or superficial structures. If the deep margin of excision is such that left anterior descending branch of the coronary artery could receive significant radiation dose, then the surface of the applicator sphere should be covered with a protective cap at the chest wall. However, in most patients the normal thickness of the chest wall (muscle and rib cage) would provide adequate shielding and such a protective cap is not required. Sometimes superficial skin flap may require protection with a 0.5cm thick, cut piece of wet gauze piece. Care must be taken however, not to inadvertently shield the areas of tissue that require radiation treatment.

IORT Dose Prescription and Delivery

The surgeon and radiation oncologist should choose the largest possible suitable applicator in order to ensure the highest dose is delivered to the tumour bed tissue. Radiation protection shielding material may also be used, which is sterile and suitable for both internal and external use.

The protocol allows for two dose prescriptions. Each participating site should decide on one method and use this procedure for all patients, for the duration of the trial.

<u>Alternative A</u>: A dose of 20 Gy at the surface of the applicator (in water) is prescribed by the radiation oncologist and delivered to the breast tissue. This takes approximately 30 minutes, depending on the size of the applicator. This prescription should be chosen if the clinic is treating mainly larger tumours (applicators ≥ 4 cm).

<u>Alternative B</u>: A dose of 6 Gy at 1 cm (in water) is prescribed by the radiation oncologist and delivered to the breast tissue. This also takes approximately 30 minutes, depending on the size of the applicator. This prescription should be chosen if the clinic is treating mainly smaller tumours (applicators ≤ 4 cm). This dose is equivalent to 5 Gy at 1 cm (adipose).

Using prescription A ensures that larger tumours receive a higher dose in the tumour bed (> 6 Gy at 1 cm and beyond). This is the reason why Alternative A is recommended for clinics with predominantly larger tumours.

Previous versions of the TARGIT protocol recommended a dose of 5Gy at 10 mm adipose which is equivalent to 6 Gy at 10 mm in water. With the publication of further research[32;33] it is now recommended that 20Gy at the applicator surface should be adopted by all new sites. This dose has superior radiobiological advantages as it ensures that tumour beds from larger tumours receive a higher dose than small tumours. Details are given in Appendix.

During the radiation treatment, the anaesthetist, clinician and physicist may remain in the room. To avoid unnecessary exposure we recommend that as many people as possible vacate the operating theatre and those remaining, either wear a lead apron or remain behind a shielded screen.

Completion of IORT

After completion of radiation, the conforming stitches are removed. Strict haemostasis should be obtained following the removal of the Intrabeam device. The skin is sutured meticulously to achieve a water-tight closure and a good cosmetic result. If non-absorbable- sutures are used, they should be left in situ for 14 days and if absorbable sutures are used, Steristrips covering the entire wound should be left in place for 14 days[23].

.

IORT Physics

Prior to entering any patient into the trial, centres will be expected to submit data for each x-ray source (XRS) probe and applicator set in use. Each centre will be responsible for measuring data for the probe and applicator set, and shall submit the data supplied by the manufacturer for comparison with measured data together with a copy of the letter of acceptance supplied by Carl Zeiss.

In addition, five "pilot" cases (non-randomised patients) will be performed followed by an audit by a member of the International Steering Committee (or an appointed delegate).

6.4.2 Conventional radiotherapy

Planning protocols for the conventional radiotherapy will vary from centre to centre but for each centre a written policy will be required (Appendix 3). All patients randomised to receive conventional radiotherapy within this trial should be treated in accordance with this policy. Dosage should only be applied to the breast; axillary, supra-clavicular and internal mammary nodes should not generally be irradiated by discrete fields. Patients with previously irradiated adjacent fields for example, those with previous contra-lateral breast cancer, will need to have the radiotherapy fields modified according to local policies. Recommended guidelines for treatment are given in Appendix.

6.5 Adjuvant Systemic Therapy

Following completion of IORT patients may be recommended appropriate adjuvant therapy according to local practice or trial protocols. For all trial patients, the sequencing of these other therapies is not governed by this protocol, but careful consideration needs to be given for patients randomised to IORT but needing external beam radiotherapy and adjuvant chemotherapy. The policy for such treatments should be declared in the Policy Statement. It is recommended that even the second procedure IORT should be delivered before beginning chemotherapy.

SECTION 7

PATIENT FOLLOW-UP AND RECORDING OF EVENTS

7.1 Follow-up and notification of recurrence, adverse events and death

Patients should be followed according to local guidelines and these should be noted on the Policy Statement prior to entry into the trial. However, case report forms will be required for each patient at 6 monthly intervals for the first five years and annually thereafter until at least 10 years.

Wherever possible each patient should be seen within a six week window of the anniversary of the therapeutic surgery (eg at 6 months ± 6 weeks). At each visit the patient should be offered a physical examination and asked whether she has experienced any adverse events.

We recommend that mammography of the ipsilateral breast occurs annually and of the contralateral breast at least every three years. Any other examination is at the discretion of the local clinician.

7.2 Recording of recurrence/new malignancies

Recurrence includes any new occurrence of breast cancer apart from new disease in the opposite breast in the absence of previous recurrence (which is classified as a contralateral breast cancer). Local recurrence, for the purposes of this protocol, is defined as recurrence in the index quadrant of the breast. Loco-regional recurrence is defined as recurrence either in the ipsilateral breast (other than the index quadrant) or in the axilla. Ipsilateral supraclavicular and intramammary node recurrence are not classified as local recurrence since the prognosis of patients with recurrence at these sites is more similar to distant than loco-regional disease.

Confirmation of ipsilateral recurrence must be by histology or unequivocal cytology. Clinical examination and imaging are not sufficient. Careful recording of the site of the recurrence is required to determine whether it is at the site of the original tumour and/or within the treatment field to determine whether the disease is ‘local’ or ‘loco-regional’.

Recurrence at any other site must be confirmed by appropriate imaging and/or biopsy. Details of both first loco-regional (breast and axilla) and first distant recurrence are required as well as the status of the patient with regard to active disease when treatment for local disease has been completed.

Development of new malignancy (including the contralateral breast) must be reported on the case report forms once a diagnosis has been confirmed.

7.3 Guidelines for the Management of Recurrence

The management of recurrent disease will be left to the discretion of the participating institution/investigator. If appropriate, patients having had IORT alone are able to have conventional irradiation as part of the management of a recurrence. It may also be possible for patients who have received external beam treatment to have IORT after surgical excision of the recurrence, particularly if they wish to avoid mastectomy.

7.4 Adverse Events

This section describes the minimum reporting procedures for the trial, based on current UK and European Union legislation. Local and national policies may require additional reporting of safety data, including adverse events.

The International Steering Committee and the DMC will review data on adverse events and complications. If a local or national policy requires a summary of this review (e.g. for an annual report), please contact the TARGIT Trial Operations Office.

Acute and late radiation morbidity must be graded according to the RTOG criteria and 'pain due to radiation' according to the Common Toxicity Criteria[34]. These are the only expected adverse events and must also be recorded on the Complications Form. Adverse events may be recorded at any time following the surgery and a new form should be completed for each episode.

7.4.1 Serious Adverse Events

A Serious adverse events (SAE) is defined as any event that is fatal; life threatening; causes or prolongs hospitalisation; causes disability or incapacity or requires medical intervention to prevent permanent impairment or damage, any grade 4 toxicity.

7.4.2 Adverse Events Reporting Requirements

Immediate reporting of any SAE must be made either directly to the International TARGIT Trial Operations Office or via the local randomisation centre as soon as possible and at least within 5 working days of the investigator becoming aware of the occurrence of the event. SAEs must be reported whether or not considered related to the treatment under investigation. An early report can be followed up by a more detailed report.

All serious adverse events that occur during the period of observation (see next section) must be documented on the 'Serious Adverse Event Form'. It is also the investigators' responsibility to report them to the local institutional ethics committee as required by local regulations.

- For all serious adverse events, the following must be assessed and recorded on the SAE reporting form: intensity/severity; relationship to treatment; action taken; outcome to date.
- Adverse events must also be recorded in the subject's medical records.

The clinical course of the serious adverse event should be managed according to accepted standards of medical practice until a satisfactory explanation is found or the investigator considers it medically justified to terminate follow-up of this event. Should the adverse event result in death, a full pathologist's report should be supplied, if possible. Any complications following surgery (± intra-operative radiotherapy) must be reported on the Complication Form.

7.4.3 Period of Observation

For the purpose of this trial the period of serious adverse event (SAE) observation extends from the time of registration onto the trial until 90 days after the completion of randomised treatment. Trial follow-up should continue according to schedule once the SAE is resolved.

7.4.4 SSARs (Suspected Serious Adverse Reactions)

Suspected Serious Adverse Reactions are adverse reactions (or events) that are thought to be related to the research procedure. For the purpose of this trial the following is a list of potential SSARs:

Fibrosis
Delayed wound healing
Wound infection
Wound breakdown
Haematoma in breast or axilla
Seroma
Dermatitis associated with radiation
Telangiectasia
Pain in irradiated field
Oedema

SSARs should be reported as described in 7.4.2

7.4.5 SUSARs (Suspected Unexpected Serious Adverse Reactions)

Suspected Unexpected Serious Adverse Reactions are adverse reactions (or events) that are thought to be related to the research procedure but do not appear on the list in 7.4.4. SUSARs should be reported as described in 7.4.2, but certainly, within 24 hours.

7.5 Deaths

All deaths, with date and cause, must be reported as soon as possible to the TARGIT Trial Operations Office on the appropriate CRF. Deaths which also meet the SAE criteria should additionally be reported as described in 7.4.2.

7.6 Withdrawal of Consent

Patients may "opt out" of the trial at any time. Rarely, patients may also wish to withdraw consent for further data collection. Such cases should be reported to the TARGIT Trial Operations Office so that no further data are entered onto the database.

SECTION 8

DATA OWNERSHIP AND PUBLICATION

The management of this trial is the responsibility of the International Steering Committee (ISC), which also owns the rights to the overall data of the trial. Individual centres own the rights to the data collected on patients entered from their own centre but by agreeing to participate in the International TARGIT Trial investigators also agree not to present or publish any analysis of the data on trial patients according to the randomised allocation until after the first publication of the overall result for the whole trial. The 'Policy for Publication of the Trial', as agreed by the International Steering Committee, is given in a separate document.

The ISC will meet regularly and review all matters of trial conduct and will also address any questions raised relating to the trial. Although Carl Zeiss Inc will be represented on the ISC, the prime responsibility for any decisions on the management of the trial will lay with the clinical and scientific academic members of the Committee.

8.1 Trial Administration

Since this trial is an international multi-centre trial it has been agreed that each centre takes responsibility for the collection and management of its own data. Randomisation can be performed centrally though the TARGIT Trial Operations Office or may be carried out locally provided the centre agrees to and abides by the 'Standard Operating Procedures for Randomisation' (available as a separate document). Several centres (e.g., in a defined geographical location) may wish to collaborate together for the randomisation process and for data collection. This is acceptable provided the arrangement is agreed by the ISC.

Definition of the data set may be determined locally provided that it accommodates the overall trial dataset as defined in the 'Data Collection Standard Operating Procedures'. Data may either be held locally and downloaded to the TARGIT Trial Operations Office as required (or at least every six months) or added directly to the trial's database via a secure website. Other methods of data entry include email, fax, and post. Each centre will be allowed only to access data on their own patients from this website. Only certain members of the ISC, the Trial Statistician and the International Project Co-ordinators will be able to access all the data. Regular electronic reports will be produced and passed to each centre for audit and checking purposes. This is a new way of managing an international clinical trial but one which allows each centre to have full responsibility for its own data.

The ISC will discuss with each centre the safeguards that are in place to secure and protect the data at their centre and also to ensure that the randomisation codes are correctly generated, stored and used for the allocation of treatments to individual patients. Failure to comply with the standards agreed by the ISC will result in all data from that centre being removed from the trial analysis.

Any data management or clinical queries should be addressed in the first instance to the Principle Investigator at the local centre. If he/she cannot adequately address the question it should be passed to the TARGIT Trial Operations Office which will either deal with the query directly or will pass it on to the co-chairs of the International Steering Committee or an agreed expert.

SECTION 9

STATISTICAL CONSIDERATIONS

9.1 Patient Numbers and Power Calculations

The objective of the trial is to determine whether the use of intra-operative radiotherapy gives equivalent rates of local control to those obtained using external beam treatment.

This is a Non-Inferiority Trial – One Sided design and we have selected a clinically meaningful non-inferiority margin $\delta_0 > 0$. We are interested in testing H_0: $\delta = \delta_0$ versus H_1: $\delta < \delta_0$ using a one-sided level-α test with power $1 - \beta$ to reject H_0 when $\delta = 0$.

We have defined the power calculations for this trial using absolute values for local breast relapse. Estimates have been obtained from published data. The CRC Trial compared the outcome of patients with good prognosis early breast cancer who had undergone breast conserving surgery and randomised to post-operative radiotherapy, demonstrated a local recurrence rate of 9% at five years in the arm treated with conventional radiotherapy. The World Overview of radiotherapy in breast cancer showed a baseline rate of about 7.8% at about 10 years. However most of these trials were started in or before 1985 and the techniques of giving radiotherapy have much improved since then such that many papers are reporting much lower rates with conventional radiotherapy.

With a baseline relapse rate of 6 % at 5 years and allowing an absolute increase or decrease in relapse rate of 2.5% (the non-inferiority margin) with 1116 patients randomised to each treatment will give a power of 80% (see Table). Thus if the recurrence rate in the IORT arm is greater than 8.5% (or lower than 3.5%) there will be an 80% chance of demonstrating a difference. If the absolute rate of recurrence is lower e.g. 5% we would have about an 85% chance of detecting a difference. This trial therefore requires a total of 2232 patients to have adequate power to meet the primary objective.

		**Power**					Confidence Interval	
	Relapse rate	**60%**	**70%**	**80%**	**90%**	**95%**	Lower	Upper
	0.03	336	438	576	797	1008	(0.005	, 0.055)
	0.04	443	578	760	1052	1330	(0.015	, 0.065)
	0.05	548	715	940	1302	1645	(0.025	, 0.075)
**P_A**	**0.06**	**650**	**849**	**1116**	**1546**	**1953**	**(0.035**	**, 0.085)**
	0.07	751	980	1288	1784	2254	(0.045	, 0.095)
	0.08	849	1108	1456	2017	2549	(0.056	, 0.105)
	0.09	944	1233	1620	2244	2836	(0.067	, 0.115)

"Statistical Methods in Medical Research" 3rd Edition p.201
P. Armitage & G.Berry, Blackwell Scientific Publications 1994

9.2 Revision of Patient Numbers

On 24 March 2010 the International Steering Committee and Data Monitoring Committee reviewed the results of an interim analysis. This showed an overall lower than expected recurrence rate at two years of 0.3% (95% CI 0.08-1.2, Kaplan-Meier estimate). A decision was made to increase accrual by an additional 1200 patients to 3432. This will also enable accrual into the sub-protocols.

9.3 Early Stopping

The DMC would keep under review the full range of adverse event data being collected and draw conclusions on safety by considering all events. If however any of the following were demonstrated at $p<0.01$ they would consider recommending early stopping:

- A significant increase in grade 4 skin or rib fractures (as a sign of radionecrosis)
- Delays in wound healing which after detailed review by the DMC as to time course and severity were considered clinically significant.

Early stopping could be applied on specific strata, while other strata are allowed to continue recruitment.

9.4 Statistical Analysis

The major endpoint is the incidence of local recurrence. This will be compared on the basis of 'intention to treat' (i.e. all randomised patients will be analysed) and the logrank test will be used. This will be performed once the baseline data have been compared to test the randomisation and to define whether any stratified analyses are required. In addition ratios of radiological lesion size to clinical and pathological size, and of specimen weight will be compared to ensure that the extent of the surgical procedure was similar in both groups. The baseline comparisons between the two groups will be done using chi-squared test for categorical variables and independent two sample t-test for all continuous variables. Statistical significance will be defined at the $p<0.05$ level. We will use Kaplan-Meier curves and proportional hazard regression models to account for time-to event and censoring of the data.

In addition, exploratory subgroup analyses will be performed on the main endpoint including variables such as tumour size, grade and axillary nodal involvement. These will be stipulated in the Statistical Analysis Plan agreed by the International Steering Committee before any analysis is undertaken.

9.5 Additional Sub-protocols

Additional sub-protocols will be written to address questions such as Quality of Life, Patient Preference, Health Economics, etc. Each sub-protocol will have its own statistical considerations.

Since we do not know what differences patients are willing to accept in recurrence rates in order to have a shorter treatment time we will be undertaking a patient preference study. This enables patients to make hypothetical 'trade-offs' between risk of relapse and treatment durations. Some of the investigators involved in TARGIT have already successfully used such studies to evaluate adjuvant hormone therapies. A separate sub-protocol will be available.

SECTION 10

ETHICAL CONSIDERATIONS

This trial, as for most randomised studies includes an experimental treatment. However, in this case the availability of the new procedure is strictly limited. There are very few machines in clinical centres and even at those centres that have the equipment, not all patients can be given the new procedure. However, should the new technique provide adequate local control and cosmesis, and be acceptable to patients it will markedly reduce the need for external beam radiotherapy for early breast cancer. This will enable a major saving of resource. The ideal time to implement a full, randomised assessment is while the technology is at fairly early stage of development. Since there are insufficient resources to give the new technique to all patients randomisation is the most ethical way to proceed. In the pilot study, every patient deemed suitable for intra-operative radiotherapy and approached consented for the procedure. We expect therefore a high acceptance of the novel arm. All suitable patients will be informed of the trial and given the opportunity to participate. Patients will be given a period (one to several days depending on the clinic timings) to consider entry and complete the consent form. Randomisation will only proceed once a signed consent form has been received at the clinic.

The trial has been planned and will be executed in line with the Declaration of Helsinki (2002).

10.1 Data Monitoring Committee (DMC)

An independent DMC has been appointed and their remit agreed. They will regularly review the data collected during the trial and report to the ISC as to whether the trial should continue and make recommendations as to changes in the protocol.

Meetings will probably be scheduled annually for the first two years of the trial whilst accrual gains momentum. More frequent meetings may then be held.

There are no formal stopping rules for the trial – these may be determined in discussion with the DMC but should a difference between the treatments in local recurrence reach $p < 0.001$ serious consideration as to whether the trial should continue will be given.

SECTION 11

QUALITY ASSURANCE

Quality assurance (QA) in radiotherapy is defined as "[the set of] procedures that ensures consistency of the medical prescription and the safe fulfilment of that prescription as regards dose to the target volume, together with minimal dose to normal tissue, minimal exposure to personnel, and adequate patient monitoring aimed at determining the end result of treatment" [35]. In the UK the Department of Health issues a mandatory standard. It is based on a formal quality management system issued by the International Standards Organisation[36]. This is a multidisciplinary responsibility to ensure consistency in absolute dosimetry, dose delivery, volume definition and reproducibility are paramount in radiotherapy QA and have become even more important with the advent of conformal therapy. Formal procedures should ensure that every "non-conformity" (i.e., a failure of any element of the system or its procedures) is identified and controlled, and that corrective action is taken to deal with the underlying causes. The quality system itself must be formally audited at regular intervals to ascertain whether it is working as intended. We expect that all investigators joining the TARGIT trial will be working to local or national standards which conform to the international guidelines. The steering committee therefore is content that no additional quality assurance exercise is necessary for conventional external beam radiotherapy provided that the investigators at each site are subject to local/national guidelines similar to those quoted above. If no such scheme operates at an investigator site the principle investigator must inform the TARGIT Operations Office who will inform the International Steering Committee to determine an appropriate course of action.

11.1 Quality assurance for the Intrabeam

All the evidence to date supports the description of the Intrabeam as delivering an accurate and reliable dose. The local physics departments are responsible for verifying the miniature X-ray source is operational prior to use, and setting up the parameters within the control console software for treatment delivery. During the procedure, the control console monitors the system for safe and accurate dose delivery.

The Intrabeam System includes a full set of quality assurance tools. These, combined with the operator interface of the control console allow complete verification of all of the performance functions in minutes and constant monitoring of critical treatment parameters throughout the treatment period. The sites are responsible for QA according to the manufacturer's instructions and these data should be made available to the trial centre. Recalibration of the output of the X-Ray Source must be performed at least annually and a report this needs to be sent to the trial centre. Carl Zeiss offers contracts for annual manufacturer service. We strongly recommend all centres to use this service. Therefore for quality assurance purposes for the TARGIT trial the Operations Office will request, annually or after every 50th patient (whichever is earlier), from each site physicist a print out of the following parameters. These will be audited by appropriate members of the steering committee and any issues will be feedback to the sites for their comment and action.

11.1.1 Parameters to be collected for QA Audit

For each trial treatment delivered the following will be collected: Applicator serial number, Applicator diameter, prescribed dose (should usually be the same in each case), treatment time, kiloVolts, and microAmps.

SECTION 12
REFERENCES

(1) Early Breast Cancer Trialists' Collaborative Group. Effects of radiotherapy and surgery in early breast cancer. An overview of the randomized trials. N Engl J Med 1995; 333(22):1444-1455.

(2) Early Breast Cancer Trialists' Collaborative Group. Favourable and unfavourable effects on long-term survival of radiotherapy for early breast cancer: an overview of the randomised trials. Lancet 2000; 355(9217):1757-1770.

(3) Clarke M, Collins R, Darby S, Davies C, Elphinstone P, Evans E et al. Effects of radiotherapy and of differences in the extent of surgery for early breast cancer on local recurrence and 15-year survival: an overview of the randomised trials. Lancet 2005; 366(9503):2087-2106.

(4) Vaidya JS, Vyas JJ, Chinoy RF, Merchant N, Sharma OP, Mittra I. Multicentricity of breast cancer: whole-organ analysis and clinical implications. Br J Cancer 1996; 74(5):820-824.

(5) Baum M, Vaidya JS, Mittra I. Multicentricity and recurrence of breast cancer [letter; comment]. Lancet 1997; 349(9046):208.

(6) Fisher ER, Anderson S, Redmond C, Fisher B. Ipsilateral breast tumor recurrence and survival following lumpectomy and irradiation: pathological findings from NSABP protocol B-06. Semin Surg Oncol 1992; 8(3):161-166.

(7) Vaidya JS, Baum M. Clinical and biological implications of the milan breast conservation trials. Eur J Cancer 1998; 34(8):1143-1144.

(8) Deng G, Lu Y, Zlotnikov G, Thor AD, Smith HS. Loss of heterozygosity in normal tissue adjacent to breast carcinomas. Science 1996; 274(5295):2057-2059.

(9) Fukino K, Shen L, Patocs A, Mutter GL, Eng C. Genomic instability within tumor stroma and clinicopathological characteristics of sporadic primary invasive breast carcinoma. JAMA 2007; 297(19):2103-2111.

(10) Vaidya JS, Vyas JJ, Mittra I, Chinoy RF. Multicentricity and its influence on conservative breast cancer treatment strategy. Hongkong International Cancer Congress 1995;Abstract 44.4.

(11) Vaidya JS, Baum M, Tobias JS, Houghton J. Targeted Intraoperative Radiothearpy (TARGIT)- trial protocol. Lancet 1999;http://www.thelancet.com/journals/lancet/misc/protocol/99PRT-47.

(12) Vaidya JS, Baum M, Tobias JS, Houghton J, Keshtgar M, Sainsbury R et al. Targeted intraoperative radiotherapy for breast cancer-a randomised trial. Breast Cancer Research and Treatment 2001; 69(3):228.

(13) Vaidya JS, Joseph D, Hilaris BS, Tobias JS, Houghton J, Keshtgar M et al. Targeted intraoperative Radiotherapy for breast cancer: An international Trial. Abstract Book of ESTRO-21, Prague 2002 2002; 21:135.

(14) Vaidya JS, Joseph D, Hilaris B, Tobias JS, Houghton J, D'Souza D et al. Targeted intraoperative radiotherapy (TARGIT) for breast cancer: an international trial. [Abstract]. Breast Cancer Research & Treatment 2002; 76 Supplement 1:S116.

(15) Joseph DJ, Bydder S, Jackson LR, Corica T, Hastrich DJ, Oliver DJ et al. Prospective trial of intraoperative radiation treatment for breast cancer. ANZ J Surg 2004; 74(12):1043-1048.

(16) Ribeiro GG, Dunn G, Swindell R, Banerjee SS. Conservation of the breast using two different radiotherapy techniques: interim report of a clinical trial. Clin Oncol (R Coll Radiol) 1990; 2(1):27-34.

(17) Ribeiro GG, Magee B, Swindell R, Harris M, Banerjee SS. The Christie Hospital breast conservation trial: an update at 8 years from inception. Clin Oncol (R Coll Radiol) 1993; 5(5):278-283.

(18) Fentiman IS, Poole C, Tong D, Winter PJ, Gregory WM, Mayles HM et al. Inadequacy of iridium implant as sole radiation treatment for operable breast cancer. Eur J Cancer 1996; 32A(4):608-611.

(19) Dale RG, Jones B, Price P. Comments on Inadequacy of Iridium Implant as sole radiation treatment for operable breast cancer, Fentiman et al.,Eur J Cancer 1996, 32A, pp 608-611. Eur J Cancer 1997; 33(10):1707-1708.

(20) Cosgrove GR, Hochberg FH, Zervas NT, Pardo FS, Valenzuela RF, Chapman P. Interstitial irradiation of brain tumors, using a miniature radiosurgery device: initial experience. Neurosurgery 1997; 40(3):518-523.

(21) Douglas RM, Beatty J, Gall K, Valenzuela RF, Biggs P, Okunieff P et al. Dosimetric results from a feasibility study of a novel radiosurgical source for irradiation of intracranial metastases. Int J Radiat Oncol Biol Phys 1996; 36(2):443-450.

(22) Vaidya JS, Baum M, Tobias JS, D'Souza DP, Naidu SV, Morgan S et al. Targeted intra-operative radiotherapy (Targit): an innovative method of treatment for early breast cancer. Ann Oncol 2001; 12(8):1075-1080.

(23) Vaidya JS, Baum M, Tobias JS, Morgan S, D'Souza D. The novel technique of delivering targeted intraoperative radiotherapy (Targit) for early breast cancer. Eur J Surg Oncol 2002; 28(4):447-454.

(24) Vaidya JS. A novel approach for local treatment of early breast cancer. PhD Thesis, University of London 2002;http://www.dundee.ac.uk/~jsvaidya/papers/thesis.htm.

(25) Vaidya JS, Baum M, Tobias JS, Massarut S, Wenz F, Murphy O et al. Targeted intraoperative radiotherapy (TARGIT) yields very low recurrence rates when given as a boost. Int J Rad Oncol Biol Phys 2006; 66(5):1335-1338.

(26) Vaidya JS, Wilson AJ, Houghton J, Tobias JS, Joseph D, Wenz F et al. Cosmetic outcome after targeted intraoperative radiotherapy (targit) for early breast caner. Breast Cancer Research & Treatment 2003; 82(S180):1039.

(27) Kraus-Tiefenbacher U, Bauer L, Scheda A, Fleckenstein K, Keller A, Herskind C et al. Long-term toxicity of an intraoperative radiotherapy boost using low energy X-rays during breast-conserving surgery. Int J Radiat Oncol Biol Phys 2006; 66(2):377-381.

(28) Kraus-Tiefenbacher U, Bauer L, Kehrer T, Hermann B, Melchert F, Wenz F. Intraoperative radiotherapy (IORT) as a boost in patients with early-stage breast cancer -- acute toxicity. Onkologie 2006; 29(3):77-82.

(29) Enderling H, Anderson AR, Chaplain MA, Munro AJ, Vaidya JS. Mathematical modelling of radiotherapy strategies for early breast cancer. J Theor Biol 2006; 241(1):158-171.

(30) Enderling H, Chaplain MA, Anderson AR, Vaidya JS. A mathematical model of breast cancer development, local treatment and recurrence. J Theor Biol 2007; 246(2):245-259.

(31) Menes TS, Tartter PI, Bleiweiss I, Godbold JH, Estabrook A, Smith SR. The consequence of multiple re-excisions to obtain clear lumpectomy margins in breast cancer patients. Ann Surg Oncol 2005; 12(11):881-885.

(32) Herskind C, Schalla S, Hahn EW, Hover KH, Wenz F. Influence of different dose rates on cell recovery and RBE at different spatial positions during protracted conformal radiotherapy. Radiat Prot Dosimetry 2006; 122(1-4):498-505.

(33) Herskind C, Steil V, Kraus-Tiefenbacher U, Wenz F. Radiobiological aspects of intraoperative radiotherapy (IORT) with isotropic low-energy X rays for early-stage breast cancer. Radiat Res 2005; 163(2):208-215.

(34) NCI. National Cancer InstituteCommon Toxicity Critaria: Radiation related adverse events and Appendix IV; RTOG/EORTC Late Radiation Morbidity Scoring Scheme. NCI 1999; [Cited 2002 June 14]:-http://ctep.cancer.gov/forms/CTCv20_4-30-992.pdf.

(35) World Health Organisation. Quality assurance in radiotherapy. Geneva 1998.

(36) International Organisation for Standardisation. Model for quality assurance in design, development, production, installation and servicing (ISO 9002). London 1994.
