# Supplementary material for: Effect of Delayed Targeted Intraoperative Radiotherapy vs Whole-Breast Radiotherapy on Local Recurrence and Survival: Long-term Results From the TARGIT-A Randomized Clinical Trial in Early Breast Cancer
Source: JAMA Oncol. 2020 Apr 2;6(7):e200249. doi: 10.1001/jamaoncol.2020.0249 (PMC7348682; doi:10.1001/jamaoncol.2020.0249)
Supplement: Supplement 2. — Data Sharing Statement [file jamaoncol-6-e200249-s002.pdf]

Data Sharing Statement

Vaidya. Effect of Delayed Targeted Intraoperative Radiotherapy vs Whole-Breast Radiotherapy on Local Recurrence and Survival. *JAMA Oncol*. Published April 02, 2020. 10.1001/jamaoncol.2020.0249

Data
Data available: Yes
Data types: Other (please specify)
Additional Information: University College London is supportive of data sharing and will endeavour to assist in requests for data sharing. All requests for data sharing will adhere to the UCL Surgical & Interventional Trials Unit (SITU) data sharing agreement policy. These data will be held at UCL on secure servers and cannot be released to any third parties. All requests for access to the data will be formally requested through the use of a SITU data request form which will state the purpose, analysis and publication plans together with the named collaborators. All requests are dealt with on a case by case basis. All requests will be logged and those successful will have a data transfer agreement which will specify appropriate security and privacy agreements, and acknowledgement of the TARGIT Trialists' Group, investigators, the sponsor, and funders.
How to access data: University College London is supportive of data sharing and will endeavour to assist in requests for data sharing. All requests for data sharing will adhere to the UCL Surgical & Interventional Trials Unit (SITU) data sharing agreement policy. These data will be held at UCL on secure servers and cannot be released to any third parties. All requests for access to the data will be formally requested through the use of a SITU data request form which will state the purpose, analysis and publication plans together with the named collaborators. All requests are dealt with on a case by case basis. All requests will be logged and those successful will have a data transfer agreement which will specify appropriate security and privacy agreements, and acknowledgement of the TARGIT Trialists' Group, investigators, the sponsor, and funders. Data queries should be made to jayant.vaidya@ucl.ac.uk
When available: beginning date: 01-31-2020, end date: 12-01-2020

Supporting Documents
Document types: None

Additional Information
Who can access the data: University College London is supportive of

data sharing and will endeavour to assist in requests for data sharing. All requests for data sharing will adhere to the UCL Surgical & Interventional Trials Unit (SITU) data sharing agreement policy. These data will be held at UCL on secure servers and cannot be released to any third parties. All requests for access to the data will be formally requested through the use of a SITU data request form which will state the purpose, analysis and publication plans together with the named collaborators. All requests are dealt with on a case by case basis. All requests will be logged and those successful will have a data transfer agreement which will specify appropriate security and privacy agreements, and acknowledgement of the TARGIT Trialists' Group, investigators, the sponsor, and funders. Data queries should be made to jayant.vaidya@ucl.ac.uk

Types of analyses: University College London is supportive of data sharing and will endeavour to assist in requests for data sharing. All requests for data sharing will adhere to the UCL Surgical & Interventional Trials Unit (SITU) data sharing agreement policy. These data will be held at UCL on secure servers and cannot be released to any third parties. All requests for access to the data will be formally requested through the use of a SITU data request form which will state the purpose, analysis and publication plans together with the named collaborators. All requests are dealt with on a case by case basis. All requests will be logged and those successful will have a data transfer agreement which will specify appropriate security and privacy agreements, and acknowledgement of the TARGIT Trialists' Group, investigators, the sponsor, and funders. Data queries should be made to jayant.vaidya@ucl.ac.uk

Mechanisms of data availability: University College London is supportive of data sharing and will endeavour to assist in requests for data sharing. All requests for data sharing will adhere to the UCL Surgical & Interventional Trials Unit (SITU) data sharing agreement policy. These data will be held at UCL on secure servers and cannot be released to any third parties. All requests for access to the data will be formally requested through the use of a SITU data request form which will state the purpose, analysis and publication plans together with the named collaborators. All requests are dealt with on a case by case basis. All requests will be logged and those successful will have a data transfer agreement which will specify appropriate security and privacy agreements, and acknowledgement of the TARGIT Trialists' Group, investigators, the sponsor, and funders. Data queries should be made to jayant.vaidya@ucl.ac.uk
